# Sustainable, scalable nanotechnology approach using filtrate from *Raphanus sativus* in combating multidrug-resistant pathogens and causing neglected tropical diseases

**DOI:** 10.3389/fcimb.2025.1684292

**Published:** 2026-01-08

**Authors:** Min Kim, Jung-Suk Sung, Seung-cheol Jee, Dae-Young Kim, Vini Mehta, Kayeen Vadakkan, Gajanan Ghodake

**Affiliations:** 1 Department of Life Science, Dongguk University-Seoul, Goyang-si, Republic of Korea; 2 Department of Biological and Environmental Science, Dongguk University-Seoul, Goyang-si, Republic of Korea; 3 Global Research Cell, Dr. D. Y. Patil Dental College & Hospital, Dr. D. Y. Patil Vidyapeeth (Deemed to be University), Pune, India; 4 Amala Integrated Medical Research Department (AIMRD), Amala Institute of Medical Sciences, Thrissur, Kerala, India

**Keywords:** silver nanoparticles, green synthesis, colloidal system, antibacterial mechanisms, multidrug-resistant pathogens, neglected tropical diseases

## Abstract

**Introduction:**

The green synthesis of silver nanoparticles (AgNPs) provides a more eco-friendly approach over the conventional chemical procedures. In this study, a fast and sustainable methodology for the production of high-density AgNPs utilizing the aqueous root filtrate of Raphanus sativus is presented.

**Methods:**

AgNPs were prepared under room temperature conditions by optimizing the concentrations of NaOH, R. sativus filtrate, and AgNO₃. UV–Vis spectroscopy was employed for characterizing AgNPs. Antibacterial properties and mechanisms of action were assessed against multi-drug resistant, gram negative Escherichia coli KCCM 11234, and gram positive Staphylococcus aureus KCCM 11335.

**Results:**

Optimally formed monodispersed AgNPs were synthesized using 0.1 mL of 1 M solution of NaOH, 1 mL (20 mM) AgNO₃ solution, and subsequent addition of plant filtrate into a final volume of 10 mL. UV-visible analysis indicated the surface plasmon resonance peak to be 405 nm, confirming the classic nucleation and isotropic growth of spherical AgNPs. The AgNPs with concentrations ranging from 20 to 30 ppm permitted the partial recovery of the bacteria and the concentrations ranging from 50 to 100 ppm showed potent antibacterial activity against MDR bacteria.

**Discussion:**

The antibacterial mechanism involved disruption of membrane integrity and permeability, leakage of intracellular substances, and oxidative damage by reactive oxygen species, resulting in bacterial cell death.

## 1 Introduction

Considerable interest has been sparked in metal NPs on account of their characteristic physicochemical properties, which include high reactivity, a high surface-to-volume ratio, tunable optical, electronic, and antibacterial properties. Such characteristics make nanoscale metal NPs (Slavin et al., 2017), oxide nanomaterials (Khater et al., 2024), and a diverse range of nanocomposites (Tang et al., 2025; Wang et al., 2025) highly multifunctional, desirable, and known for their satisfactory applications in nanomedicine fields (Meher et al., 2024), agriculture (Wahab et al., 2024), plant growth (Khan et al., 2023), and environmental fields (Mo et al., 2022). Among such metals, silver (Ag) NPs have been in wide usage owing to their low cost and unique physicochemical properties, contributing to improvements in a wide range of consumer products and biomedical formulations (Haider and Kang, 2015; Kailasa et al., 2019). However, despite immense potential for the commercial application of AgNPs, conventional techniques such as physical and chemical techniques are being used for their production (Ferdous and Nemmar, 2020). The process utilizes toxic reagents, is energy-intensive, and produces byproducts, which is alarming due to health concerns and environmental impact (Iravani et al., 2014). Therefore, greener and environment-friendly synthesis methods need to be explored, which can achieve industry-scale demand with a maximum of biocompatibility and less environmental implications (Sharma et al., 2022).

Green fabrication of metal NPs is an environment-friendly and safer strategy to the conventional process. It takes advantage of sustainable biological resources like fungi (Talebian et al., 2023), algae (Chan et al., 2022), and plant extracts (Rani et al., 2023; Tabasum et al., 2025). These entities are rich in wide ranges of biomolecules that act like natural capping and reducing reagents, and therefore it is ideal to be employed in the preparation of metal NPs under nontoxic and mild conditions. Among microbes, various fungal and bacterial strains have so far been well documented in the literature and regarded as biogenic methods. All of these methods have their own merits and demerits (Dhaka et al., 2023). Several species of both microalgae and macroalgae (seaweed) have so far been employed in the green synthesis of AgNPs (Choudhary et al., 2024). Biosynthesis of the NPs can be either intracellular or extracellular; special attention is focused on the latter extracellular biosynthesis due to the simplicity of the purification and NP recovery (Tończyk et al., 2023). Microbial synthesis of metal NPs is a greener approach compared to many of the existing chemical and physical methods; however, it is not without its own challenges, which involve cultivation, harvest, and downstream processing, which are time- and labor-intensive and require specialized conditions of cultivation. This may lead to problems in reproducibility or scaling up (Liu et al., 2024).

On the other hand, the simple, scalable, and cost-effective use of plant extract for nanosynthesis has introduced it as a popular approach toward green nanotechnology (Shafey, 2020; Tabasum et al., 2022).​ Low-cost, accessible, and watery root vegetable *R. sativus* is nutritionally rich (Kumar et al., 2022) and consists of a wide range of bioactive phytochemicals (Gazizova et al., 2025; Park et al., 2022), including amino acids, organic acids (Cai et al., 2024), flavonoids (Zhang et al., 2020), polyphenols (Goyeneche et al., 2015), glucosinolates (glucoraphasatin) (Yan et al., 2023), terpenes, and antioxidants (Gamba et al., 2021). They are best suited to decrease metal ions to metal NPs and concurrently stabilize them to prevent aggregation (Sidhu et al., 2022). In this paper, we describe the rapid production of AgNPs using radish root (*R. sativus*) filtrate and demonstrate a green method for the preparation of high-concentration suspensions of colloidal AgNPs. This study abides green chemistry principles; it employs green solvents and mild reaction conditions and also avoids the use of toxic reagents or minimizes waste byproducts (Dahl et al., 2007; García-Quintero and Palencia, 2021).

*R. sativus* filtrate-mediated protocol for AgNP synthesis seems to be facile, rapid, scalable, and sustainable. A clear filtrate free of suspended solids is proposed as a reducing and stabilizing agent for the rapid, safer, and facile synthesis of colloidal AgNPs under mild conditions (24°C). We investigated the effect of various reaction constraints on the yield, optical properties, and stability of AgNPs in aqueous media. A potent antibacterial action of AgNPs against multidrug-resistant *S. aureus* and *E. coli* via the loss of membrane integrity and oxidative stress is demonstrated. Although an intrinsically active or potent antibacterial agent, caution is essential because of the possibility of developing resistance.

## 2 Materials and methods

### 2.1 Materials

The chemicals, namely, sodium chloride, silver nitrate, Gram’s iodine reagent, glutaraldehyde, poly-L-lysine, and crystal violet solution, were acquired from Sigma-Aldrich (www.sigmaaldrich.com). Standard solutions of NaOH and phosphate buffer solutions (10 J of pH 7.4) were obtained from Daejung Chemicals, Korea (www.daejungchem.co.kr). Yeast extract, peptone, nutrient broth powder, and agar powder were supplied by Becton Dickinson Chemicals. Multidrug-resistant (MDR) strains of *S. aureus* [KCCM 11335] and *E. coli* [KCCM 11234] were obtained from the Korean Culture Center of Microorganisms (KCCM), Seoul, South Korea. Deionized water was freshly prepared using a Thermo Scientific system and was used for reagent formulations, reaction mixtures, and AgNP synthesis. All assays were performed in triplicate to validate reproducibility, and values are presented as mean ± standard deviation.

### 2.2 Preparation of *R. sativus* filtrate

Fresh *R. sativus* root vegetables of similar size and maturity from the local market in Seoul, South Korea, were collected for the preparation of the aqueous filtrate. The green tops and the root ends were cut off, followed by unpeeling the skin to normalize the sample. Then, these were sliced into even-sized pieces of around 1 to 2 cm and homogenized in a laboratory blender. Thereafter, centrifugation (12,000 rpm, 10 min) was carried out, and the supernatant was sensibly collected. The mixture was filtered through Whatman paper to eliminate the suspended solid particles. The colorless, clear filtrate was stored in amber glass bottles in a refrigerator at 5°C.

### 2.3 AgNP synthesis and purification

AgNPs were prepared using the metal precursor, silver nitrate (AgNO_3_), and the *R. sativus* root aqueous filtrate in alkaline condition at room temperature (24°C). To examine the synthetic method, we studied the influence of AgNO_3_ (0.5, 1.0, 2.0, 3.0, 4.0, and 5.0 mM), *R. sativus* filtrate (0.125, 0.25, 0.5, 1.0, and 2.0 mL), and NaOH (0.5, 1.0, 2.0, 3.0, 4.0, and 5.0 mM) concentrations on the yield of AgNP and their optical properties. The total volume of all reactions was maintained to be about 10 mL. ​ AgNP synthesis performance of the green procedure was monitored by taking 1 mL of AgNPs and recording the UV–vis spectra using a UV–vis spectrophotometer (Optizen-2120). ​AgNP synthesis at optimum settings was done in 10 mL of reaction mixture containing 1 mL *R. sativus* filtrate with 0.1 mL 1 M NaOH and the subsequent addition of 1 mL of 20 mM AgNO_3_ solution to start the reaction. Following biosynthesis at optimal condition, the AgNPs were purified through ultracentrifugation (12,000 rpm, 15 min, 15°C). The supernatant was safely discharged, and the subsequent pellets were dispersed in cold deionized water to eliminate excess NaOH, unreacted Ag ions, and residual biomolecules. The purified AgNPs were stored at room temperature for additional characterization done systematically as well as antibacterial mechanism determination. All AgNP preparation trials were conducted three times for reproducibility, and values were expressed as mean ± standard deviation.

### 2.4 Characterization of AgNPs

The physicochemical characterization of the AgNPs in the current study was analyzed by means of different advanced methods, such as X-ray diffraction (XRD), Fourier transform infrared spectroscopy (FTIR), high-resolution transmission electron microscopy (HR-TEM), X-ray photoelectron spectroscopy (XPS), and zeta potential measurements. The AgNP thin films were drop-casted onto clean and dry glass substrates, air-dried at 60°C, and characterized using a Cu–Kα radiation (PANalytical X’Pert Pro) XRD instrument. The FTIR spectra of both filtrate and purified AgNPs were recorded in transmission mode using an ATR arrangement of a FTIR spectrometer using Nicolet iS50, Thermo-Fisher Scientific (Waltham, MA, USA). The stability of AgNPs prepared at diverse concentrations of NaOH was examined by zeta potential after the ratio of AgNPs/DI water (0.2:0.8) was diluted by ELSZ-2000 (Otsuka Electronics). HR-TEM imaging and structural analysis by selected area electron diffraction (SAED) pattern of AgNPs were carried out using JEOL JEM-2100 to examine the shape, size, morphology, and composition of AgNPs. XPS survey spectra and narrow scans were achieved to characterize the oxidation states of surface elements in AgNPs with an angle-resolved theta probe (Thermo-Fisher Scientific, Waltham, MA, USA). The *in vitro* stability of AgNPs was studied by maintaining the solution in DI water for 30 days at ambient temperature. The conditions observed for UV–vis spectra were monitored periodically at 3-day intervals.

### 2.5 Antibacterial studies

Antibacterial assessment of AgNPs against MDR *S. aureus* and *E. coli* was determined using microbial assays (*n* = 3). Freshly grown culture of a bacterium in nutrient agar plate at 37°C were inoculated into the nutrient broth and grown in shaking condition at 120 rpm. The newly cultured bacterial cells had a final density of ~10^5^ CFU/mL by suspending in PBS buffer (50 mM, pH 7.4). The nutrient broth that was sterilized was inoculated with either *E. coli* or *S. aureus* and contained the AgNP suspensions at concentrations of 20 and 200 ppm. The cultures were incubated in a shaking incubator (120 rpm) at 37°C, and the growth was examined by measuring the optical density and minimum inhibitory concentration (MIC) at 600 nm using an absorbance microplate reader (TECAN, Männedorf, Switzerland) (Parvekar et al., 2020). The spread plate method was used to find out the least concentration of the AgNPs by determining the minimum bactericidal concentration (MBC) (Parvekar et al., 2020). The treated bacterial cultures (50, 100, and 200 ppm AgNPs) were spread onto nutrient agar plates. The minimum bactericidal concentration was taken as the lowest concentration of AgNP that inhibited the growth of all bacterial colonies after 24 h of incubation at 37°C.

The morphological effect of AgNPs on *E. coli* and *S. aureus* was analyzed by using optical and field emission scanning electron microscopes (FE-SEM). Bacterial cells exposed to 30 ppm of AgNPs for 2 h were pelleted at around 3,700 rpm for 12 min (*n* = 3), washed with phosphate-buffered saline (PBS, 1×, pH 7.4), and imaged. For optical microscopy, bacterial suspensions were washed away, stained with 1% crystal violet and Gram’s iodine solution, and counterstained with safranin. The heat-fixed slides were rehydrated with DI water, and images were taken to profile bacterial size, shape, and form. For FE-SEM imaging, bacteria that were loaded on poly-L-lysine-coated coverslips for 2 h at 37°C were treated with 50 ppm AgNPs, fixed in glutaraldehyde (2.5%) at 4°C for 24 h, and dehydrated in serial alcohol concentrations (30%, 40%, 60%, 80%, or 100%) for 10 min each. The bacterial samples were platinum-sputtered and observed under FE-SEM (Hitachi S-4700, Tokyo, Japan).

The disruption of the bacterial membrane was evaluated by the release of bacterial proteins into culture supernatants by the bicinchoninic acid (BCA) protein assay kit (Thermo Fisher Scientific, Waltham, MA) according to the manufacturer’s instructions. Briefly, bacteria were treated with a sub-lethal amount of AgNPs for a given period of time and then allowed to grow under normal conditions. Following treatment, the cultures were centrifuged at 10,000 × *g* for 10 min at 4°C to obtain the supernatant. The supernatant protein contents of the trials were determined by 25 μL of each sample in 200 μL/well of BCA working reagent (prepared as per the manufacturer’s instructions) in 96-well plates for 30 min at 37°C, examined by absorbance reading at 562 nm on a microplate reader. Protein leakage was normalized to cell viability by conducting a separate series of 10‐fold dilutions, plating on LB agar (*n* = 3), and counting colonies after overnight incubation at 37°C, and the values are expressed as microgram of protein released/10–^6^ viable cells.

Intracellular ROS measurements were conducted using 2′,7′-dichlorofluorescein diacetate (DCFDA; Sigma-Aldrich, Burlington, MA, USA). In brief, bacterial cultures treated with a sub-lethal dosage of AgNPs were collected (*n* = 3), washed twice with phosphate buffer, and then raised with 10 μM DCFDA in PBS at 37 °C in the dark for 30 min. After staining, the cells were washed free of excess dye and exposed to test compounds under cell culture conditions. Immediately after treatment, the fluorescence (excitation at 485 nm, emission at 535 nm) was examined by using a microplate reader (BioTek Instruments, Winooski, VT, USA). To adjust for differences in bacterial viability, CFUs were determined in parallel by plating serial dilutions on Luria–Bertani agar and incubating them overnight at 37°C. The ROS levels were normalized to viable cell numbers and expressed as relative fluorescence units per 10^6^ CFU (RFU/10^6^ CFU).

Assessment of bacterial resistance to AgNP was determined using 24-h bacterial cultures grown in tryptic soy broth (TSB, CA, USA) at 37°C with shaking. The bacteria were diluted to OD600 = 1.0, and then the bacteria were exposed to a sub-lethal dosage of AgNP for 4 h. After treatment, bacterial growth was determined as OD_600_; an aliquot of the culture was subsequently incubated in fresh TSB without AgNP overnight. This routine was carried out daily: regrowth to OD_600_ = 1.0, 4 h AgNP treatment, OD600 measurement, and subculturing overnight. Bactericidal effectiveness was determined at each cycle in order to track changes in the susceptibility of the bacteria throughout time.

Mouse embryonic fibroblast (MEF) cells were obtained from the American Type Culture Collection (Manassas, VA, USA). MEF cells were cultured in DMEM (Gibco, NY, US) supplemented with 10% FBS (Gibco, NY, USA), 100 μg/mL streptomycin, and 100 U/mL penicillin (P/S, Gibco, USA) in a 100-mm^2^ culture dish. The culture medium was replaced with fresh medium every second day without any additional interventions. The MEF cells were preserved in the incubator with the condition of 37°C and 5% CO_2_. Cytotoxicity was measured by using EZ-CYTOX reagent (DOGEN) according to the manufacturer’s protocol. A normal MEF cell line was seeded onto 96-well plates at 1 × 10^4^ cells/well, and AgNP was treated at various concentrations. The used medium was substituted with freshly prepared medium including EZ-CYTOX reagent 24 h after incubation. The plates were read at 450 nm by employing a microplate reader (Molecular Devices, San Jose, CA, USA) after 2 h of incubation. The cell viability of AgNP-treated cells were matched with control groups.

### 2.6 Statistical analysis

Three replicates (*n* = 3) were performed in this experiment. Data were statistically processed and presented as mean ± SEM. Statistical analysis was performed using one-way ANOVA and Tukey’s post-test. Data analysis was conducted with SigmaPlot 10.0 and GraphPad Prism 5.0 (GraphPad Software Inc.). Results with *p <*0.05 were deliberated as statistically significant.

## 3 Results and discussion

### 3.1 Synthesis of AgNPs

All the AgNP preparation reactions were conducted in triplicate at room temperature (24 °C). The formation and growth of AgNPs were monitored by UV–vis spectra between 5 and 60 min. A sharp surface plasmon resonance (SPR) band of AgNPs at 405 nm was used to demonstrate the effective synthesis and stabilization. The SPR peak started to appear at 5 min and was further continued to increase in intensity, representing the rapid nucleation and growth of AgNPs (**Supplementary Figure S1a**). Such a rapid synthesis process was verified by the highest-intensity peak, which appeared in a short period, indicating efficient NP formation. The SPR peak further sharpened and slightly increased until 30 min, signifying simultaneous NP formation and stabilization. The stationary phase appeared at 60 min when the SPR peak reached maximum intensity, indicating the completion of the formation and growth of AgNPs. This distinct SPR peak indicates that rapid nucleation and yielding of AgNPs can be efficiently accomplished with this green method. The rapid synthesis of AgNPs can be attributed to the presence of different phytochemicals in the *R. sativus* filtrate that acts as both the reductant toward a rapid reduction of Ag ions and the stabilizing agent responsible for protecting the formed AgNPs (Chandrasekharan et al., 2022; Kazemi et al., 2023).

It should be emphasized that after 60 min, the SPR peak of the AgNP colloid attained an optical density of approximately 2.0 (**Supplementary Figure S1b**). It puts forward that the Ag ion reduction by *R. sativus* aqueous filtrate forms a clear and strong SPR peak that is also indicative of very good colloidal stability, a narrow size distribution, and a lack of aggregation. These optical properties also indicate the formation of uniformly dispersed, monodisperse AgNPs with high prospective for antimicrobial activity and catalysis. The efficacy of this room temperature synthesis protocol, without the use of man-made chemicals, follows the numerous principles of green chemistry, particularly in terms of energy efficiency and environmental sustainability (O’Neil et al., 2021).

**Figure 1:**
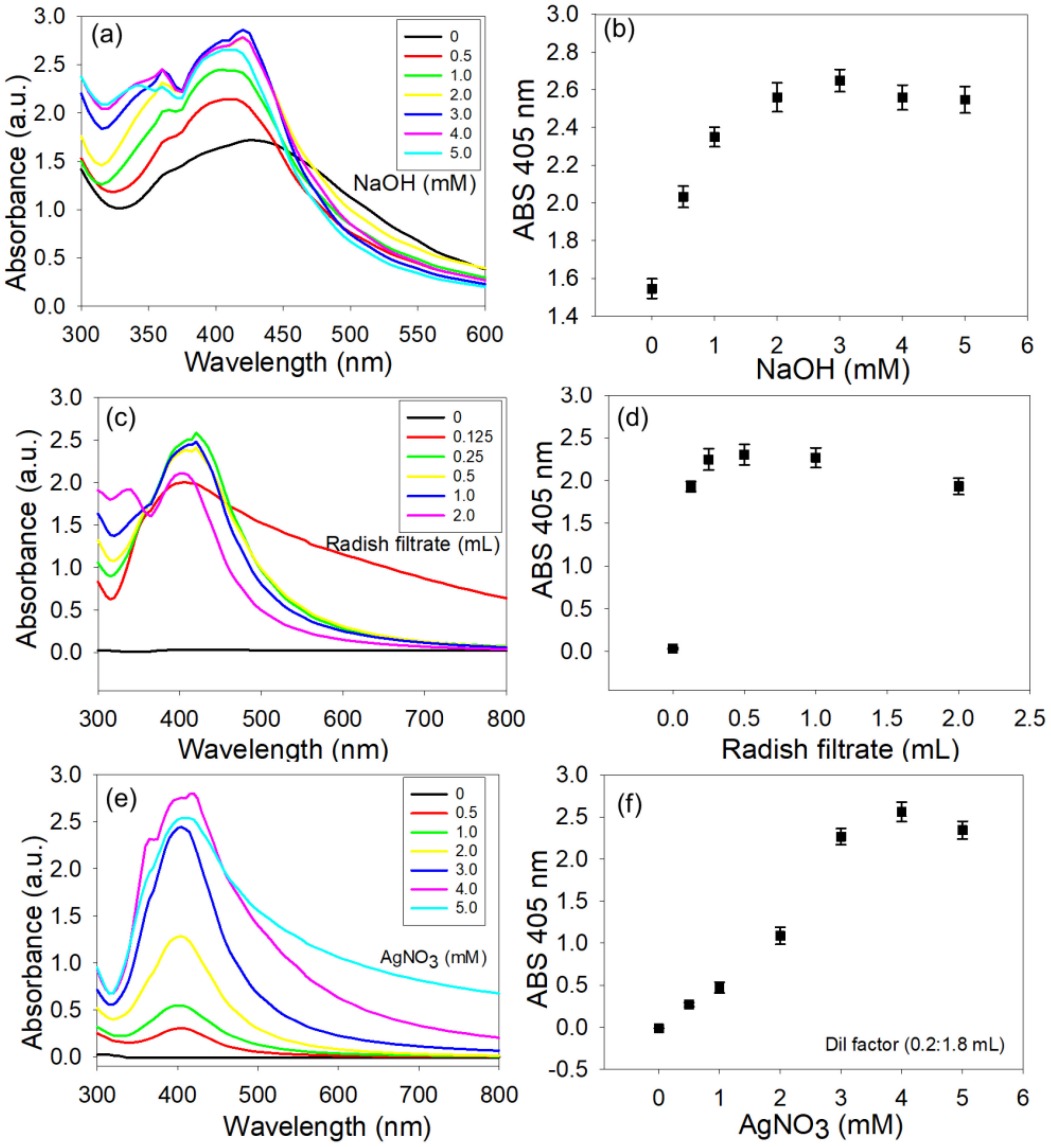
UV–vis spectral and quantitative analyses showing the influence of key synthesis parameters on AgNP formation. **(a, b)** Effect of NaOH concentration on the surface plasmon resonance (SPR) band, demonstrating improved intensity and linear correlation with particle yield. **(c, d)** Effect of *Raphanus sativus* filtrate concentration in a 10-mL reaction mixture, indicating enhanced AgNP formation with increasing phytochemical content. **(e, f)** Effect of AgNO_3_ precursor concentration on SPR band profile and absorbance at 405 nm, confirming its direct dependence on AgNP yield.

UV–vis spectroscopy was further used to confirm that the synthesis of AgNPs by the *R. sativus* filtrate is strictly dependent on the concentration of NaOH in the reaction medium. In the absence of NaOH, AgNP formation was ineffectual, showing a broad and weak SPR band at the representative AgNP absorption area (**Figure 1a**). Such a broad SPR band indicates that NPs have a broad size distribution and a poor yield, which typically comes from the slow and insufficient reduction of Ag^+^ ions. As the concentration of NaOH increased, a pronounced change in the UV–vis spectra was found with a narrowing trend for SPR bands and an increase in the intensity values (**Figures 1a, b**). The decreasing broadness of the SPR band specifies a monodisperse, narrow size distribution and shows that the AgNPs prepared at alkaline are more uniform in size. The alkaline condition prepared by the addition of the NaOH would probably upsurge the deprotonation (Aigbe and Osibote, 2024) and reactivity of the functional groups of phytochemicals present in the *R. sativus* extract. This facilitates the electron transport progressions associated with the reduction of Ag^+^ to Ag^0^, which leads to a more homogenous and faster nucleation (Gangwar et al., 2021). Faster kinetics reduce secondary nucleation, growth, and aggregation in solution, manufacturing smaller and more homogeneously dispersed NPs (Iravani, 2011).

In addition, there is an increasing trend of the SPR peak intensity with the NaOH concentration (**Figures 1a, b**). This optical density surge is directly proportional to an increase in the concentration of AgNPs in the colloidal solution, which means that the production of NPs can be controlled well in an alkaline medium. The data highlights the importance of the alkaline environment for the facilitation of green synthesis of AgNPs. The increase in the peak intensity also indicates that effective deprotonation of functional groups plays a major role by increasing the reducing power of the bioactive phytochemicals and successfully stabilizing the colloidal suspensions of AgNPs through capping interaction. The alkalinity of the reaction mixture efficiently facilitates AgNP synthesis and institutes favorable situations for rapid nucleation and uniform growth as well as the enhancement of the NP’s yield as noted in a previous report (Singh et al., 2009).

From a mechanistic viewpoint, the existence of a well-defined and narrow SPR peak in the UV–vis spectra confirms that phytochemicals from *R. sativus* sufficiently cap the colloidal NPs and protect them from aggregation. **Supplementary Table S1** presents a list of the bioactive components of *R. sativus* that include flavonoids (38.8%), polyphenols (8.4%), terpenoids (8.2%), glucosinolates (5.6%), isothiocyanates, antioxidants, biopolymers (starch and pectin), and amino acids (Gamba et al., 2021) that have reactive functional groups such as carbonyl (C=O), hydroxyl (–OH), and carboxyl (–COOH) groups (Hanlon and Barnes, 2011). The deprotonated functional groups of these molecules are involved in both reducing Ag^+^ ions to Ag^0^ and adsorbing onto the surfaces of AgNPs, thus forming a protective organic capping and ensuring excellent colloidal stability (Oselusi et al., 2025).

The newly formed AgNPs are thermodynamically unstable and have a tendency to aggregate and agglomerate owing to their high surface energy, wherein phytochemicals in *R. sativus* play a key role via diverse mechanisms (Fernando and Zhou, 2019). The predominant mechanism is electrostatic stabilization, involving deprotonated –OH and –COOH groups of polyphenols or phenolic acids, which provides a net negative charge at the surface of NPs and subsequently repulsive interactions (Mahajan et al., 2025). Natural polysaccharides like pectin and starch can play a key role in providing steric stabilization by starting a spatial shield around the NPs that can limit the interactions of adjacent NPs (Luo et al., 2021). Flavonoids like kaempferol and quercetin are exceptional antioxidants with multi-hydroxy functionalities, useful to encourage effective Ag^+^ reduction composed with good capping onto AgNP surfaces (Goyeneche et al., 2015; Zhang et al., 2020). Moreover, *R. sativus* is a good source of amino acids, peptides, and proteins that likely act as reducing as well as stabilizing agents, having the property to provide steric hindrance through their amine and thiol functionalities (Mei et al., 2022; Vriens et al., 2016).

UV–vis spectroscopy was also used as an analytical tool to monitor the formation, growth kinetics, and optical properties of the AgNPs fabricated using different concentrations of *R. sativus* filtrate. Typically, in the lower concentration of the filtrate, the UV–vis spectra showed a broad and weak SPR band in the region of 405 to 420 nm (**Figure 1c**). The broad SPR peak suggests that the AgNP size is quite diverse, the nucleation is not even, and Ag^+^ ions are not completely reduced. This is predictable when the concentration of phytochemicals in the solution was not sufficient to fully reduce the precursor Ag ions or to stabilize the nucleated crystals sufficiently. At a concentration of 0.25 mL of *R. sativus* filtrate in a 10-mL reaction volume, a distinct, strong, sharp SPR peak was observed (**Figure 1c**), indicating the development of monodisperse and well-stabilized AgNPs. Such a SPR peak suggests that the tuning reaction conditions can result in homogeneity of the narrow size distribution, and the intensity of the peak is a measure of the density of the AgNPs. These finding suggests that 0.25 to 0.5 mL can be the optimum volume of the filtrate to achieve the equilibrium of reducing agents and capping agents and to result in both maximum nucleation proficiency, atom economy, and colloidal stability.

This observation can also be documented by the trend of absorbance intensity at λmax, a characteristic wavelength of approximately 405 nm, of the SPR results from the spherical AgNPs. Absorbance displays the highest intensity value at 0.25, 0.5, and 1.0 mL of filtrate (**Figure 1d**), suggesting that the concentration of *R. sativus* filtrate plays a key role in AgNP production. Notably, when a higher concentration of filtrate (2.0 mL) was used, the SPR peak intensity was reduced (**Figure 1d**). Since the absorbance is directly relative to the concentration, the higher peak intensity reflects a higher density of well-formed AgNPs (Shukla et al., 2008), which also ratifies that the method is effective and suitable for the quantitative reduction of Ag^+^ ions. A reproducible optical signal obtained in the recurring experiments is a good proof for the superior steadiness and reliability of biosynthetic AgNP production using *R. sativus* filtrate. A previous paper presented a novel, surfactant-free, and energy-efficient technique for the synthesis of AgNPs using an aqueous extract of *Zingiber officinale* rhizome as a reducing and stabilizing agent in conjunction with Ag precursor solution (Prasad et al., 2023).

The potential of *R. sativus* filtrate in the green synthesis of AgNP was monitored across a range of AgNO_3_ precursor concentrations to assess its ability for controlled formation and high-yield production. To ensure precision and consistency in the spectral measurements, a standardized dilution procedure was involved, wherein the as-prepared AgNP solution was diluted with DI water at a ratio of approximately 0.2:1.8 mL. The resulting AgNP dispersions were planned at different AgNO_3_ concentrations ranging from 0.5 to 5.0 mM using UV–vis spectroscopy. A strong dependence of the NP yield on increasing AgNO_3_ concentration was witnessed from the UV–vis spectral analysis (**Figure 1e**). The UV–vis spectra of AgNPs at lower to moderate AgNO_3_ concentrations (0.5–3.0 mM) showed sharp, narrow SPR bands located at around 405 nm corresponding to well-dispersed, spherical AgNPs with narrow size distributions. Notably, SPR intensity improved steadily with increasing AgNO_3_ concentration in this range, proving the sustainability and suitability of the method to achieve high AgNP yield. These findings also demonstrate the excellent effectiveness of the *R. sativus* process in the reduction of Ag ions into AgNPs with better optical characteristics and colloidal stability, whereas at higher precursor concentrations (4.0 and 5.0 mM), noteworthy broadening and red-shift of the SPR bands were seen (**Figure 1e**), indicative of higher polydispersity, broad size distribution, and possible aggregation (Sangaonkar et al., 2020). These results also display a saturation point, i.e., where the amount of the bioactive molecules in the *R. sativus* filtrate is not enough to completely reduce surplus Ag ions and stabilize AgNPs, causing uncontrolled growth and size variation (Ansari et al., 2023).

The quantitative analysis of AgNP formation was piloted for AgNPs prepared with different AgNO_3_ concentrations (0.5 mM to 5.0 mM) using *R. sativus* filtrate. To maintain measurement consistency, AgNP samples were diluted in a constant ratio (0.2 mL AgNPs/1.8 mL DI water) before analysis. At this 10-fold dilution, the UV–vis spectra showed high absorbance intensities, particularly for 0.5–3.0 mM AgNO_3_, confirming the synthesis of high-concentration colloidal AgNP suspensions (**Figure 1f**). Using a higher precursor concentration (4.0 and 5.0 mM) resulted in broad SPR bands, signifying more polydisperse and less colloidal stability. Nonetheless, the high absorbance intensities indicate that a comparatively high proportion of AgNO_3_ was reduced to the consistent AgNP concentrations, suggesting that the method is suitable for high-throughput synthesis. The data highlight the high atom economy of the *R. sativus*-assisted process, in which the majority of Ag ions are converted to AgNPs with negligible byproduct formation. A high efficiency consistent with the principles of green chemistry allows the careful use of natural resources and the mitigation of nano-waste generation (Anastas and Zimmerman, 2003; Hutchison, 2008). The high yield, uniform size distribution, and high extinction coefficient together indicate the scalability and possible applications of this green synthesis procedure, a deliberate effort toward sustainable nanomaterial manufacturing. Similar observations been reported in studies about the synthesis of physiologically stable AgNPs using *Zingiber officinale* extract, which demonstrated an efficient green synthesis approach that produces bioactive functionalized AgNPs possessing the ability to act against *Bacillus subtilis* (Prasad et al., 2023).

### 3.2 Characterization of AgNPs

To examine colloidal stability, the UV–vis spectra, bandwidth (Δλ), and resonance maximum wavelength (λmax) of AgNPs were measured after each 3rd day of the AgNP solution, as shown in **Supplementary Figure S2**. Absorbance at λmax (405 nm) was insignificantly affected with the incubation time. While the absorbance slightly decreased, Δλ and λmax did not change, which indicates that the structural and colloidal stability of the AgNPs is excellent even after 30 days of incubation, so there is no concern on stability change or antibacterial activity. The AgNP suspension in DI water showed stable UV–vis absorption peak at 405 nm, confirming its good colloidal stability. In biomedical scenarios where nanoparticles are colloidal, it is important to test the stability of AgNPs on storage. As mentioned in previous sections, stability studies were performed for AuNPs (Shukla et al., 2008).

**Figure 2:**
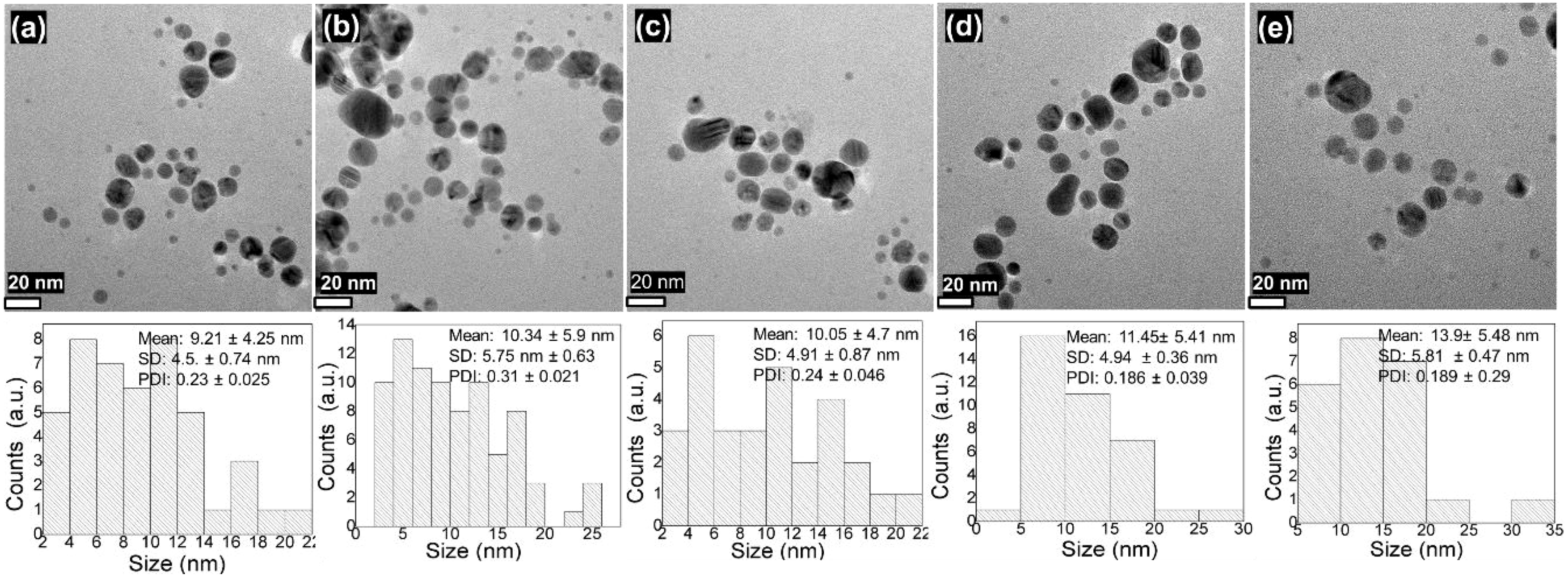
**(a–e)** HR-TEM images of AgNPs collected at various NaOH concentrations (0.5 to 4.0 mM) with spherical to hexagonal well-dispersed AgNPs and the respective size distribution histogram with average mean size, SD, and PDI values.

Such stability was further confirmed by observing for aggregation using HR-TEM images and validating good surface capping and avoiding aggregation or oxidation, which support the *in vitro* stability of AgNPs. HR-TEM was used to determine the morphology, size distribution, and dispersion of AgNPs under different synthesis conditions. The HR-TEM images of the reaction’s final product at different NaOH concentrations (0.5–4.0 mM) indicated that the AgNPs were predominantly spherical, whereas some oval to hexagonal-like structures were also found at certain concentrations of NaOH (**Figures 2a–e**). The AgNPs seemed to be well separated and discrete without any pronounced agglomeration, suggesting good colloidal stabilization. A quantitative grain size examination from the HR-TEM images indicated that the mean NPs’ size varied from 9.21 to 13.9 nm, depending on the NaOH concentration. The size variability among particles in each of the samples was of a relatively low standard deviation (SD = 4.5–5.8 nm). Moreover, the achieved polydispersity index (PDI) values were in the range of 0.18–0.30, indicating a reasonably narrow size distribution, and monodisperse. These results show the well-controlled, consistent, and reproducible nature of AgNPs’ growth under different alkaline conditions as obtained via *R. sativus* filtrate-mediated synthesis. The identical size and shape of the AgNPs (**Figures 2a–e** and **Supplementary Figure S3a**) are ascribed to the dual role of phytochemicals as good reducing and stabilizing agents in enabling fast nucleation and controlled particle growth (Kordy et al., 2022). Furthermore, the typical crystalline structure of the prepared AgNPs was also confirmed by the SAED pattern, where well-defined concentric bright rings for (111), (200), (220), and (311) planes reveal the face-centered cubic (fcc) lattice crystalline structure of the AgNPs (**Supplementary Figure S3b**). These results were additionally confirmed by XRD, indicating sharp peaks at specific 2θ values corresponding to the fcc AgNPs. The strong and well-defined SAED patterns and XRD peaks reflect good crystallinity, signifying again the good quality and stability of the biosynthesized NPs.

**Figure 3:**
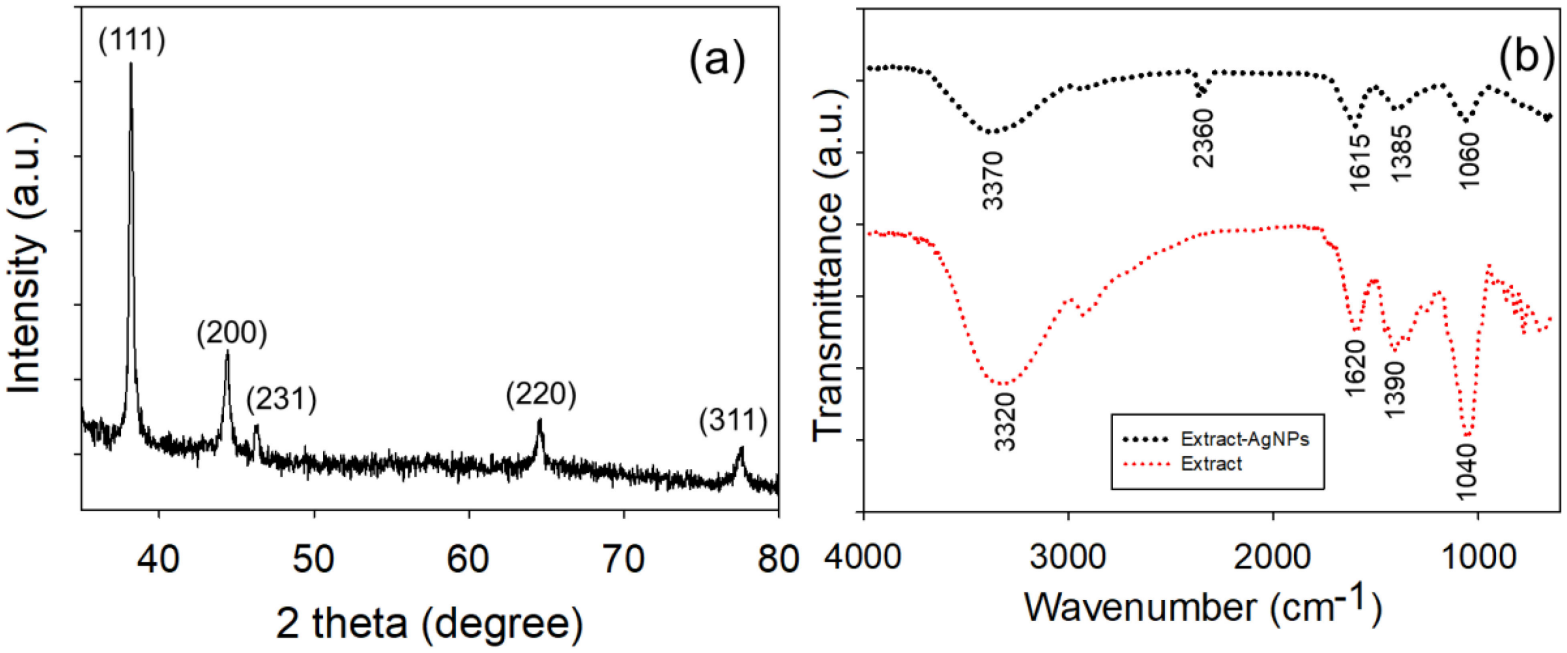
**(a)** XRD patterns of AgNPs displaying distinct peaks corresponding to the (111), (200), (220), and (311) planes, confirming the fcc crystalline structure of AgNPs. **(b)** FTIR spectra of *R. sativus* filtrate and AgNPs, highlighting functional groups such as C=O, O–H, C–N, and C–O, which are involved in reducing Ag ions and stabilizing AgNPs.

The XRD pattern was determined to confirm the crystalline nature, phase purity, and average crystallite size of AgNPs prepared from *R. sativus* filtrate. Four well-specified and intense peaks were detected in the diffraction pattern at 2θ values of 38.11°, 44.32°, 64.53°, and 77.34°, which were indexed as the diffraction of (111), (200), (220), and (311) plane attributes to the fcc structure of metallic AgNPs, respectively (Garibo et al., 2020). These reflections correspond to those of standard data in the Joint Committee on Powder Diffraction Standards (JCPDS), to which these were compared, and the JCPDS card no. 04-0783, which showed the synthesized material as a crystalline elemental of Ag, as shown below in **Figure 3a**. Sharp, well-defined XRD peaks signify the high crystallinity and phase purity of spherical AgNPs (Esther Arland and Kumar, 2024). The results are in agreement with the SAED pattern of sharp lattice fringes and diffraction rings of identical fcc structures (Manzoor et al., 2024). The Scherrer equation, as stated in previous reports, was applied to the full width at half-maximum of the dominant (111) peak to evaluate the average crystallite size (Ali et al., 2023). Using this approach, the average crystallite size of the AgNPs was determined to be approximately 14–18 nm, depending on the NaOH concentration used in the synthesis. These sizes are consistent with the sizes determined by HR-TEM, indicating the nanometer-scale sizes and single-crystalline nature of the particles. This investigation suggests that the production of crystalline AgNPs is significant in areas where crystal structure affects performance—for example, in the context of antibacterial electronic applications, high crystallinity enhances surface reactivity and long-term stability (Sunil et al., 2021). The results indicate that the hydrothermal synthesis conditions significantly affect the structural, optical, and antibacterial properties of CeO_2_ nanoparticles and clearly point to the direct relationship between physicochemical characteristics and biological activity, especially antibacterial properties (Gajbhiye et al., 2024).

The bioreduced and stabilized AgNPs using *R. sativus* filtrate were characterized by FTIR spectroscopy to elucidate the functional groups accountable for the reduction of Ag ions and formation of AgNPs. The FTIR spectrum of AgNPs displays distinctive absorption peaks corresponding to the different biomolecular functional groups of the phytochemicals (**Figure 3b**). A wide and intense band was seen at around 3,320 cm^−1^ owing to the stretching vibrations of hydroxyl groups (O–H), which are typically present in polyphenols and flavonoids (Pielorz et al., 2023). A strong band at approximately 1,600 to 1,620 cm^−1^ is often assigned to the stretching vibrations of the C=O double indicating carbonyl groups in organic molecules, comprising those from carboxylic acid (Parlinska-Wojtan et al., 2016). In addition, absorption peaks at 1,400 and 1,050 cm^−1^ were ascribed to C–N stretching (amines or amides) and C–O stretching (phenolic acid, flavonoid, protein, and polysaccharides), respectively. The presence of these vibrational bands reveals that several types of phytochemicals (including flavonoids, phenolic compounds, terpenoids, and, to a lesser extent, proteins) are involved in reducing the Ag^+^ ions to metallic Ag^0^ and capping agents that help to stabilize the AgNPs. These biomolecules may provide electrons via surface redox-active functional groups (e.g., hydroxyl or carbonyl) to Ag^+^ ions to initiate nucleation and, at the same time, protect the AgNP surface from aggregation by steric and charge repulsion (Huo et al., 2019). The FTIR spectra are in good agreement with the described literature related to similar spectral bands in a green synthesis system; the –OH, C=O, and aromatic groups play a main role in the synthesis and capping of AgNPs (Parlinska-Wojtan et al., 2016). In addition, the slight peak broadening or disappearance for the post-synthesis sample designates that these groups are involved in capping the AgNP surface, contributing to colloidal stability and uniform distribution (Pasieczna-Patkowska et al., 2025). In a previous report, XRF, FTIR, and GC–MS analyses have shown that flowers from 20 herbaceous plants have differences in a rich profile of bioactive chemicals, thus showing their value for resource utilization.

XPS was employed for the surface elemental purity and chemical state analysis of AgNPs produced using *R. sativus* filtrate. The XPS survey spectrum (**Supplementary Figure S4a**) showed the signals of Ag, O, and C, which showed the existence of oxygen and carbon from the phytochemicals of *R. sativus* extract, which act as capping and stabilizing agents, and Ag originates from the metal core reduction. The high-resolution XPS spectra of Ag showed two peaks, which were attributable to Ag 3d_3_/_2_ and Ag 3d_5_/_2_ at approximately 374.3 and 368.2 eV, respectively (**Supplementary Figure S4b**). The ~6.1-eV splitting of this spin–orbit doublet corresponds closely to a previously reported value of metallic Ag^0^, suggesting a successful reduction of Ag^+^ ions to their “zero-valent” state and indorsing the pure metallic nature NPs (Kim et al., 2024). No additional peaks or shoulders were observed in the Ag 3d region, indicating minor oxidation or Ag oxide formation and stabilization of the NPs in their metallic form (Vinoth et al., 2017).

The result of a high-resolution O 1s spectrum was a peak at 532.0 eV (**Supplementary Figure S4c**), which is commonly assigned to oxygen in hydroxyl (–OH) and carbonyl (C=O) groups. Such functional groups are characteristic of flavonoids, phenolic acids, and other biomolecules and most likely involve the formation and stabilization of the AgNPs. It also indicates the surface interaction of AgNPs with the organic matrix, which can also contribute to the resistance to oxidation by passivating the AgNP surface. As shown in **Supplementary Figure S4d**, the peak for the C 1s spectrum could be separated into two main peaks at 284.8 and 286.2 eV, which could be attributed to the C–C/C–H and C–O/C=O bonding states, respectively. The identification of these functional groups also agrees well with the FTIR spectrum, which indicates the participation of *R. sativus* phytochemicals in the surface capping and colloidal stabilization of the NPs.

Zeta potential examination was performed to evaluate the surface charge and colloidal stability of AgNP suspension prepared with *R. sativus* filtrate. The average zeta potential value was approximately –35.6 ± 7.0 mV (**Supplementary Figure S5**), which suggests that AgNPs displayed a strongly negative surface charge. The probable presence of negative charges can be due to the adsorption of negatively charged phytochemicals (anionic phytochemicals like phenolic acids, flavonoids, carboxylated biomolecules, etc.) over the surface of AgNPs. In general, a high absolute zeta potential value (higher than ±30 mV) is considered to be a good hint of electrostatic stability in colloidal systems. The negatively charged surface facilitates repulsion among the AgNPs, so the aggregation is repelling and long-term dispersion stability is assured in aqueous solvents. These results match well with the HR-TEM and UV–vis results showing well-dispersed NPs without any signs of agglomeration. This value of surface potential supports the involvement of the *R. sativus* phytochemicals in both the reduction (of Ag^+^ to Ag^0^) and capping and stabilization of the AgNPs. This organic corona of the AgNPs has a negative charge that stabilizes the colloidal suspensions of AgNPs. Such inherent stability is a critical factor for further applications in the biomedical, antimicrobial, and environmental areas, which require a long dispersion and a shelf life. It has been designated in the literature that for AgNPs to demonstrate colloidal stability, the zeta potential values should be generally a minimum of ±30 mV in absolute value (Yadav and Preet, 2023).

### 3.3 Antibacterial studies

**Figure 4:**
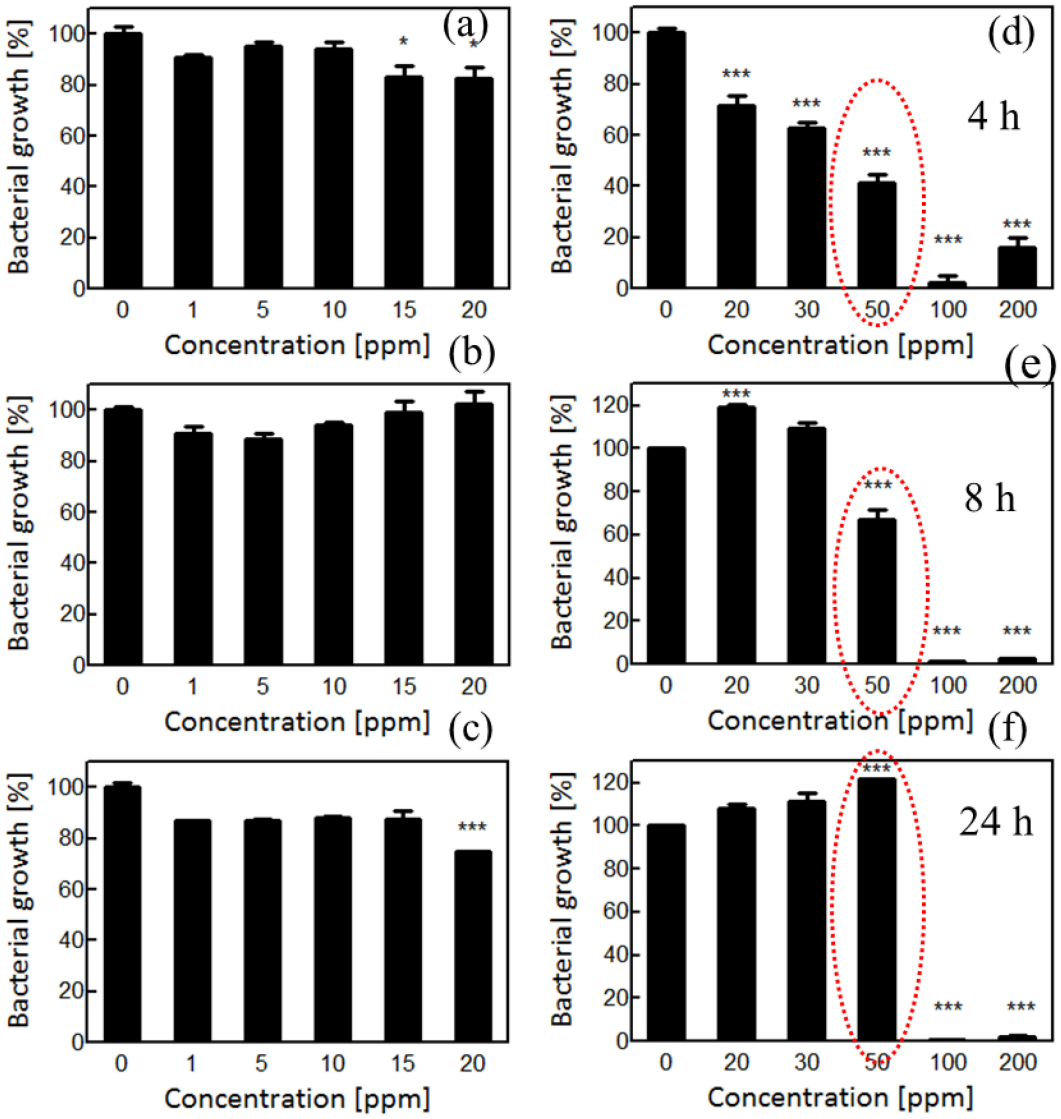
**(a–c)** Effect of low AgNP concentration ranges on *S. aureus* growth at different time intervals. **(d–f)** Effect of high AgNP concentration ranges on *S. aureus* growth at different time intervals. Significant differences are marked with asterisks.

The antibacterial potential of AgNPs synthesized using *R. sativus* filtrate was assessed for the MDR strains of *E. coli* and *S. aureus*. These two bacterial organisms have very different cell wall structures. *S. aureus* has a thick peptidoglycan layer (Wang et al., 2022) without an outer membrane, while *E. coli* features an outer membrane with lipopolysaccharides (Mikheyeva et al., 2023).These structural differences are critical in determining the permeability and antimicrobial susceptibility of AgNPs. A broad range of AgNP concentrations (1–200 ppm) was tested to assess dose-dependent antibacterial effects. At low concentrations (1–20 ppm), minimal bacterial growth inhibition was observed during all growth phases (**Figures 4a–c**). This suggests that the sub-threshold AgNP doses did not adequately penetrate into the bacterial cell envelope or disrupt intracellular processes. The *Piper Longam* extract exhibited MIC and MBC values of 0.5–1.0 mg/mL against *Escherichia coli* O157:H7, and at ½× MIC it produced 66%–75% inhibition of biofilm formation (Wiriyaprom et al., 2025).

At a moderate AgNP concentration (20–50 ppm), a clear inhibition of early bacterial growth was evident, particularly during the lag phase (within 4 h, **Figure 4d**), indicating bacteriostatic effects. This effect is probably mediated through membrane interaction, enhanced membrane permeability (Sun et al., 2016), ATP synthesis inhibition (Wypij et al., 2021), and interference with metabolic essential enzymes (Rodrigues et al., 2024). Remarkably, a partial recovery of *S. aureus* growth at 8–24 h with 20–30 ppm AgNPs (**Figure 4e**) suggests the activation of adaptive stress responses. The recovery could be a result of the activation of the stress response pathway to attain tolerance of sub-lethal AgNP exposure over time; such multifaceted mechanisms have been formerly described in bacteria (Hochvaldová et al., 2024). However, adaptive phenomena may allow subpopulations to survive sub-lethal AgNP stress. On the other hand, at 100 ppm, the AgNPs exerted bactericidal activity and entirely inhibited the growth of the bacteria (**Figure 4f**). This is attributed to extensive membrane damage, loss of membrane integrity, cytoplasmic leakage, and cell lysis (Zharkova et al., 2021).

**Figure 5:**
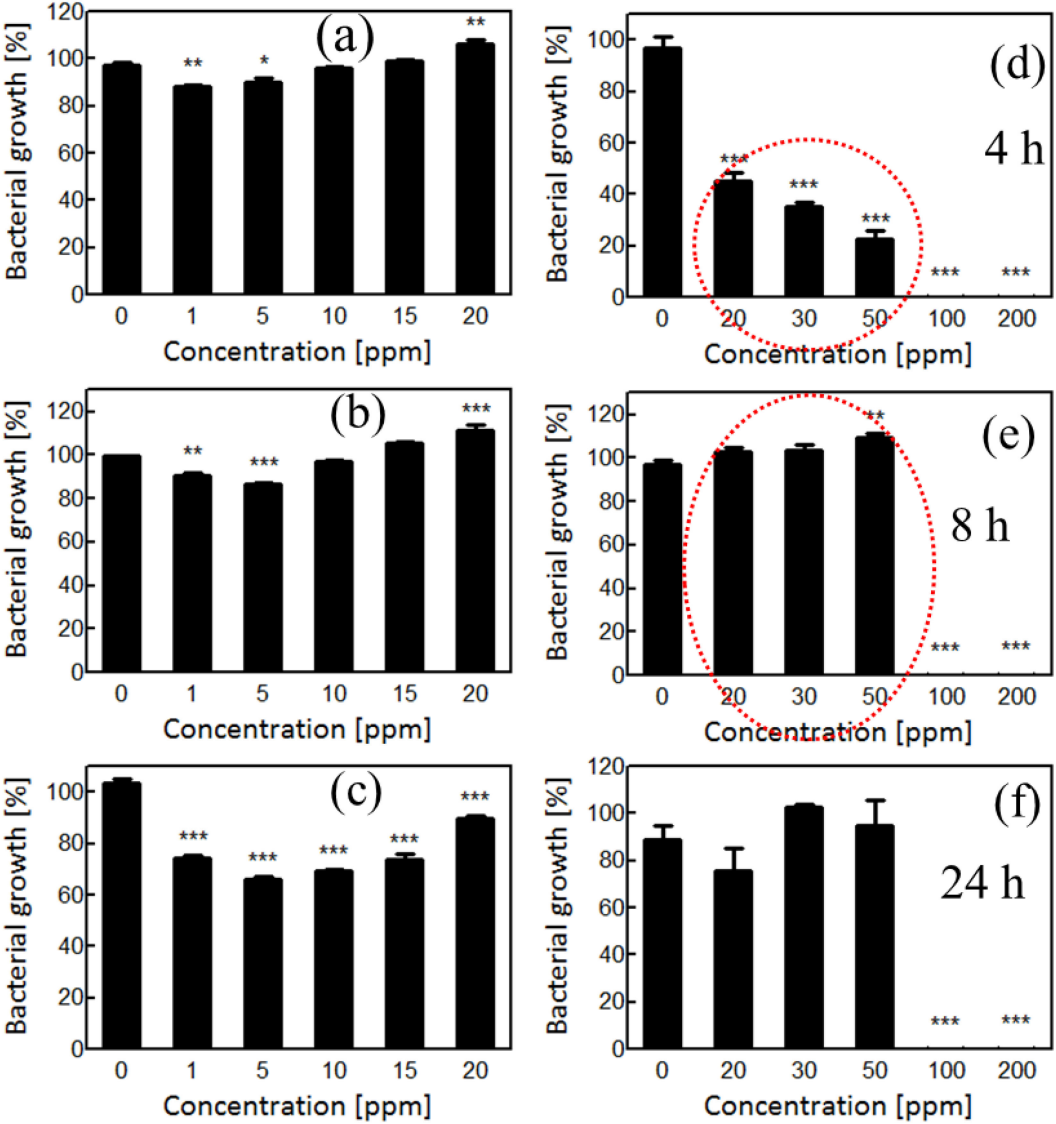
**(a–c)** Effect of low AgNP concentration ranges on *E. coli* growth at different time intervals. **(d–f)** Effect of high AgNP concentration ranges on *E. coli* growth at different time intervals. Significant differences are marked with asterisks.

In *E. coli*, a similar trend was observed. Low concentrations (1–20 ppm) produced negligible antibacterial effects (**Figures 5a–c**), likely due to the protective nature of the Gram-negative outer membrane. Moderate concentrations (20–50 ppm) inhibited growth early in the exponential phase (**Figure 5d**), but a recovery in viability by 8 h (**Figure 5e**) suggests an effective stress adaptive mechanism, possibly involving AgNP sequestration or encapsulation by a subset of the population (Li et al., 2010). The subset of cells may encapsulate or sequester the AgNPs and die but, in doing so, protecting the majority. This collective stress response ensures the survival of the population of bacteria and subsequent proliferation on return from the acute threat of the AgNPs. At 100 ppm, AgNPs displayed a strong bactericidal activity against *E. coli* (**Figure 5f**), likely multiple mechanisms, including ROS generation (Quinteros et al., 2016), interference with DNA and thiol-containing proteins (Ezeuko et al., 2022), oxidative damage (Naik and Ramana Devi, 2021), and cell death (Campo-Beleño et al., 2022). The *E. coli* with the outer membrane is more vulnerable than the thick cell wall of *S. aureus* to the entry of AgNPs, leading to more bacterial cell death at the early growth stages (Dat et al., 2021; Nikolic and Mudgil, 2023). The action sites of AgNPs in Gram-negative bacteria are considered to be outer membranes (Nie et al., 2023), wherein AgNPs potentially interact with lipopolysaccharide components (Wu et al., 2022).

Colony-forming unit (CFU) counting is a direct measure of the number of viable bacterial cells that can multiply directly, which is extensively adopted to evaluate the effectiveness of antimicrobial agents (Balouiri et al., 2016). CFU counts also indirectly suggest the potential ability of bacteria to form biofilms in the future. Biofilms pose a serious public health threat, especially in the case of chronic and recurring infections, medical device contamination, and water-borne diseases (Zhao et al., 2023). Biofilms of MDR-resistant pathogens are developing to be extremely resistant to conventional antibiotics and immune system responses. In the current study, the antibacterial activity of AgNPs synthesized with *R. sativus* filtrate was tested quantitatively by using CFU assays on both *S. aureus* and *E. coli*. A significant viability loss in both species for both tested bacteria was observed at 50 ppm of AgNPs. There was an estimated reduction in CFU count of 73.6% for *E. coli* (**Supplementary Figure S6a**), whereas the bactericidal activity against *S. aureus* showed an even greater reduction of approximately 86.68% under the same conditions (**Supplementary Figure S6b**). Furthermore, the results at 100 ppm show an extraordinary reduction in the viability of the bacteria strains, emphasizing the promising bactericidal activity of the AgNPs and their being a promising agent against biofilm-associated bacteria.

Gram-stain micrographs and high-resolution FE-SEM of *S. aureus* and *E. coli* treated with 50 ppm of AgNPs revealed structural damage and morphological changes. In untreated *S. aureus*, a Gram-positive bacterium appeared as clusters of round, purple cells due to retention of crystal violet in the thick peptidoglycan layer. However, such coccal clusters seem poorly organized in the post-treatment images (**Supplementary Figures S7a, b**). The treated *S. aureus* cells show abnormalities in shape and disturbed boundaries, and it was observed that the cell wall integrity was damaged, which clearly indicates that the AgNPs could penetrate through the thick peptidoglycan (**Supplementary Figure S7b**). More evidence of cytoplasmic leakage, cell debris, and cell wall disruption are clearly visible with compromised cell integrity (**Supplementary Figure S7b**). In the case of *E. coli*, a Gram-negative bacillus, an untreated cell appeared pink because the crystal violet is easily washed from the thin layer of peptidoglycan, allowing safranin to be retained. The rod-like shape of *E. coli* cells was found to be significantly impaired in the treated cultures (**Supplementary Figures S7c, d**).

**Figure 6:**
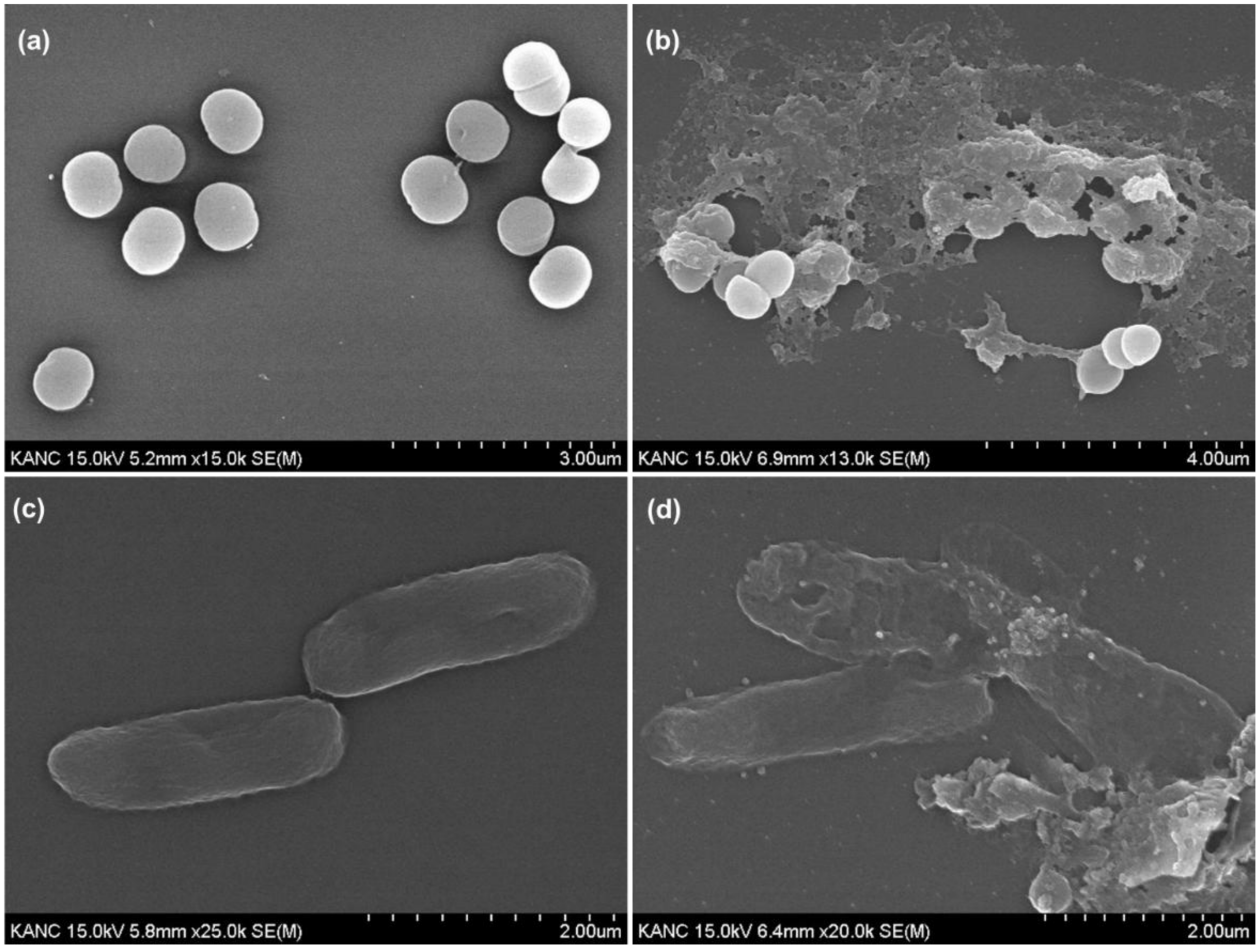
FE-SEM imaging of bacterial cells, **(a)** non-treated *S. aureus*, **(b)** AgNP-treated *S. aureus*, **(c)** non-treated *E. coli*, and **(d)** AgNP-treated *E. coli*.

FE-SEM analysis further supported these findings. Untreated *S. aureus* cells were smooth and round with intact membranes (**Figure 6a**), while treated cells exhibited membrane collapse, ruptures, visible cracks, and debris, indicating peptidoglycan damage and subsequent cytoplasmic leakage (**Figure 6b**). The drastic structural changes are consistent with previous findings (Zhang et al., 2022). For *E. coli*, the untreated cells had a rod-shaped structure with a smooth outer membrane (**Figure 6c**), but treatment led to deformation, fractures, and membrane roughening (**Figure 6d**). The observed damage is caused by AgNP interactions with the outer membrane, especially their strong affinity for the lipopolysaccharide layer (Bondarenko et al., 2018) and the resulting disruption of the membrane integrity (Xiao et al., 2021). Furthermore, the loss of the cell wall integrity results in the release of intracellular material, resulting in bacterial cell death (Yakoup et al., 2024), which is further supported by the protein leakage and ROS measurement.

**Figure 7:**
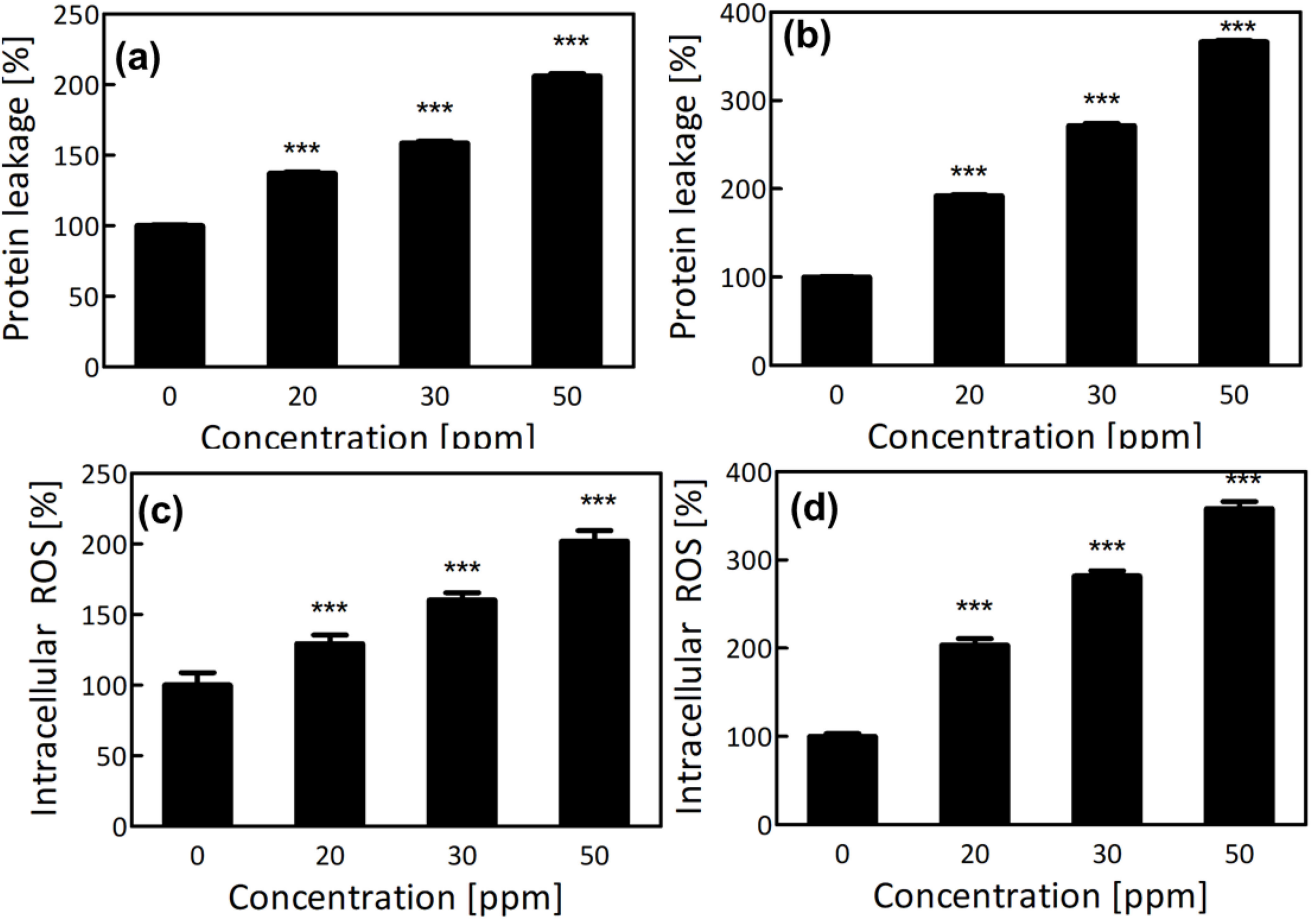
**(a)** Percentage of protein leakage in *E. coli* following AgNP treatment. **(b)** Percentage of protein leakage in *S. aureus* following AgNP treatment. **(c)** Percentage of ROS generation in *E. coli* induced by AgNPs. **(d)** Percentage of ROS generation in *S. aureus* induced by AgNPs. Significant differences are marked with asterisks.

The antibacterial action of AgNPs in specific relation to cell membrane damage and intracellular oxidative stress was extensively studied using *E. coli* and *S. aureus*. A dose-dependent increase in extracellular protein release was noticed, with the highest release at 50 ppm (**Figures 7a, b**) corresponding to the FE-SEM observations of widespread membrane disintegration. The leakage was indicative of a loss of selective permeability and damage to the cytoplasmic membrane, the early lethal effect of AgNPs on bacterial cells (Dakal et al., 2016). The physical damage would also be in accordance with earlier reports that the AgNPs’ interaction with membrane phospholipids and surface proteins results in the disruption of membrane structure and cell lysis (Singhal et al., 2024). Both bacteria exhibited pronounced protein leakage, likely due to the interaction of AgNPs with both the peptidoglycan layer and negatively charged lipopolysaccharides (Ferreyra Maillard et al., 2019).

Moreover, the antibacterial activity of *R. sativus*-synthesized AgNPs appears to involve a synergistic mechanism, combining physical membrane disruption with oxidative stress. AgNP exposure led to dose-dependently elevated ROS generation, including O_2_^−^, •OH, and H_2_O_2_, peaking at 50 ppm (**Figures 7c, d**). These reactive species cause lipid peroxidation, protein oxidation, DNA damage, and enzyme inactivation cascading into cell membrane destabilization, intracellular leakage, and eventual cell death (Dakal et al., 2016; Qing et al., 2018). This dual-mode antibacterial mechanism structural disruption and intracellular oxidative stress render AgNPs highly effective and less prone to resistance development. Unlike conventional antibiotics that target specific enzymes or pathways, AgNPs exert broad-spectrum, non-specific oxidative damage, making it more difficult for bacteria to evolve resistance (Hochvaldová et al., 2024; Wang et al., 2021). These findings highlight the promising applications of green-synthesized AgNPs as potent antimicrobial agents, particularly against multidrug-resistant bacteria.

We further examined the potential for bacterial adaptation and resistance to the synthesized AgNPs by performing serial passage assays with *E. coli* and *S. aureus* under sub-lethal AgNP concentrations. *E. coli* showed gradual restoration of viability with each subsequent transfer (**Supplementary Figure S8a**), suggesting an adaptive response potentially involving physiological and/or genetic changes that confer increased tolerance to AgNP-induced stress (Graves et al., 2015). These findings align with previous reports on bacterial responses to the metal-based antimicrobials (More et al., 2023). Repeated low-dose AgNP exposure has been shown to increase the minimum inhibitory concentration and alter bacterial surface properties, indicative of tolerance or resistance development (Stabryla et al., 2021). Additionally, non-genetic resistance mechanisms such as flagellin overproduction, which aggregates and neutralizes AgNPs, have been observed in *E. coli* and *Pseudomonas aeruginosa* (Panáček et al., 2018).

In contrast, *S. aureus* did not exhibit notable recovery in viability during the assay period (**Supplementary Figure S8b**), suggesting variability in resistance development between bacterial species. While short-term adaptation was absent, long-term sub-lethal exposure may still promote resistance in *S. aureus* through mechanisms like metal sequestration, gene regulation, or biofilm-associated tolerance. Both *E. coli* and *S. aureus* have been reported for their ability to activate efflux pumps, changes in membrane lipid profile, and induction of identical oxidative stress-responsive pathways upon exposure to AgNPs (Hochvaldová et al., 2024) or synergistic effect of antibiotics and AgNPs (Nefedova et al., 2022). These findings raise concerns about the potential for resistance development due to the uncontrolled use of nanomaterials. Thus, there is an increasing need to adopt stringent nano-waste management practices, controlled disposal routines, and well-organized eco-toxicological examinations (Kamat and Kumari, 2023). Such assessments need to advance beyond acute toxicity studies and also take into account long-term exposure effects, such as microbial adaptation potential in the field environment and clinical ecosystems (Kaweeteerawat et al., 2017).

### 3.4 Environmental application feasibility and future prospective

The comparative biological effects of the prepared AgNPs were studied as a function of cytotoxicity by MEF cell lines. As illustrated in **Supplementary Figure S9**, no significant cytotoxic effect was detected with exposure to AgNP over the entire concentration range applied to the cells, showing good biocompatibility as reported in a previous report (Khare et al., 2022). High cell viability was retained, with no significant change in the morphology of cells or metabolic impairment, indicating that the AgNPs are well tolerated by MEF cell lines in the provided experimental conditions. These results suggest that the AgNPs are mildly toxic to mammalian cells and can be safely used for future applications in the biomedical or environmental fields at lower concentrations effective for their antimicrobial activity. It should be noted that the excessive presence of AgNPs in the environment could result in bioaccumulation, food chain adulteration, and resistance in pathogenic microorganisms (Mat Lazim et al., 2023). Thus, controlled use, environmentally safe disposal, and careful consideration of long-term effects on the ecosystem is prerequisites for safe and sustainable use of AgNPs in environmental systems (Kakakhel et al., 2021).

The green production of AgNPs using *R. sativus* filtrate is cost-effective and environmentally benign compared to chemical methods. The phytomediated extracellular procedure has the advantage of reducing Ag ions and stabilizing AgNPs without using toxic agents and follows green chemistry processes, minimizing waste and by-products. The biosynthetic AgNPs are well known to be with excellent multifunctional properties for antibacterial purposes. Notably, the AgNPs were highly bactericidal toward MDR strains, including *E. coli* and *S. aureus*, suggesting their applications as potential agents for treating microbial-contaminated water, industrial effluents, and medical tools.

## 4 Conclusions

Herein we report an economical and environmentally sustainable process for the green synthesis of AgNPs mediated by the aqueous filtrate of *R. sativus*. The synthesis process very well depends on plant phytochemicals, greener agents for efficiently reducing Ag ions, and stabilization of AgNPs. The synthesized AgNPs possessed good physicochemical properties such as relatively narrow size distribution, colloidal stability, and antibacterial activity, which can be readily used in practical applications. Antibacterial studies suggest that the prepared AgNPs strongly suppressed the growth of MDR strains of *E. coli* and *S. aureus* by increasing bacteria membrane permeability, intracellular leakage, and intracellular oxidative stress. Although the results emphasize the positive therapeutic potential of AgNPs, the concerns over bacterial resistance at sub-lethal doses also suggest that optimization of dosing and nano-waste management practices is necessary to limit adaptation. Further work is necessary for the scale-up synthesis of AgNPs and long-term evaluation of the environmental and biological effects.

## Data Availability

The original contributions presented in the study are included in the article/**Supplementary Material**. Further inquiries can be directed to the corresponding author.
