# Supplementary material for: Sustainable, scalable nanotechnology approach using filtrate from Raphanus sativus in combating multidrug-resistant pathogens and causing neglected tropical diseases
Source: Front Cell Infect Microbiol. 2026 Jan 8;15:1684292. doi: 10.3389/fcimb.2025.1684292 (PMC12823997; doi:10.3389/fcimb.2025.1684292)
Supplement: Supplementary file 1 [file Table1.docx]

**Sustainable, scalable nanotechnology approach using filtrate from Raphanus sativus in combating multidrug-resistant pathogens and causing neglected tropical diseases**

**Running Title: Sustainable green nanotech using *Raphanus sativus* against MDR and NTD pathogens**

Min Kim^1^, Jung-Suk Sung^1^, Seung-cheol Jee^1^, Dae-Young Kim^2^, Vini Mehta^3^, Kayeen Vadakkan^4^, and Gajanan Ghodake^2^*

^1^Department of Life Science, Dongguk University-Seoul, Biomedical Campus, 32 Dongguk-ro, Ilsanadong-gu, Goyang-si 10326, Gyeonggi-do, South Korea

^2^Department of Biological and Environmental Science, Dongguk University-Seoul, Biomedical Campus, 32 Dongguk-ro, Ilsanadong-gu, Goyang-si 10326, Gyeonggi-do, South Korea

^3^Global Research Cell, Dr. D. Y. Patil Dental College & Hospital, Dr. D. Y. Patil Vidyapeeth (Deemed to be University), Pimpri, Pune 411018, India

^4^Amala Integrated Medical Research Department (AIMRD), Amala Institute of Medical Sciences, Thrissur, Kerala, 680555, India

*Corresponding author: Gajanan Ghodake; Email: ghodakegs@dongguk.edu; Tel: +82-31-961-5159; Fax: +82-31-961-5122


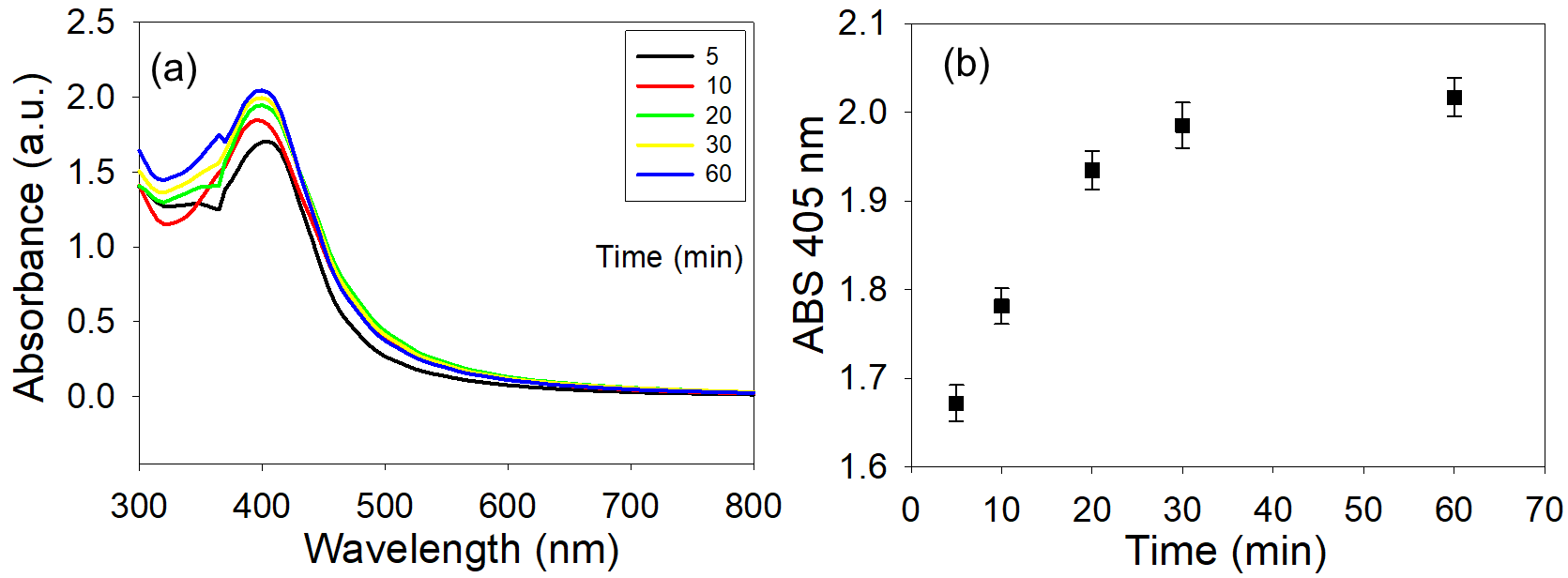


**Figure S1.** (a) UV-Vis spectra of AgNPs recorded at different time intervals, (b) Peak intensity of SPR bands as a function of time.


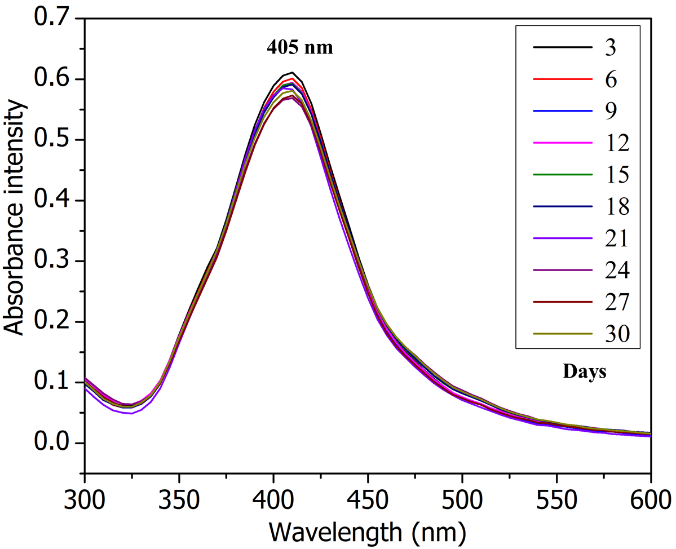


**Figure S2.** The stability of the prepared AgNPs has been confirmed using monitoring UV-vis spectrums for 30 days for the colloidal dispersion efficacy and the aggregation risk factor.


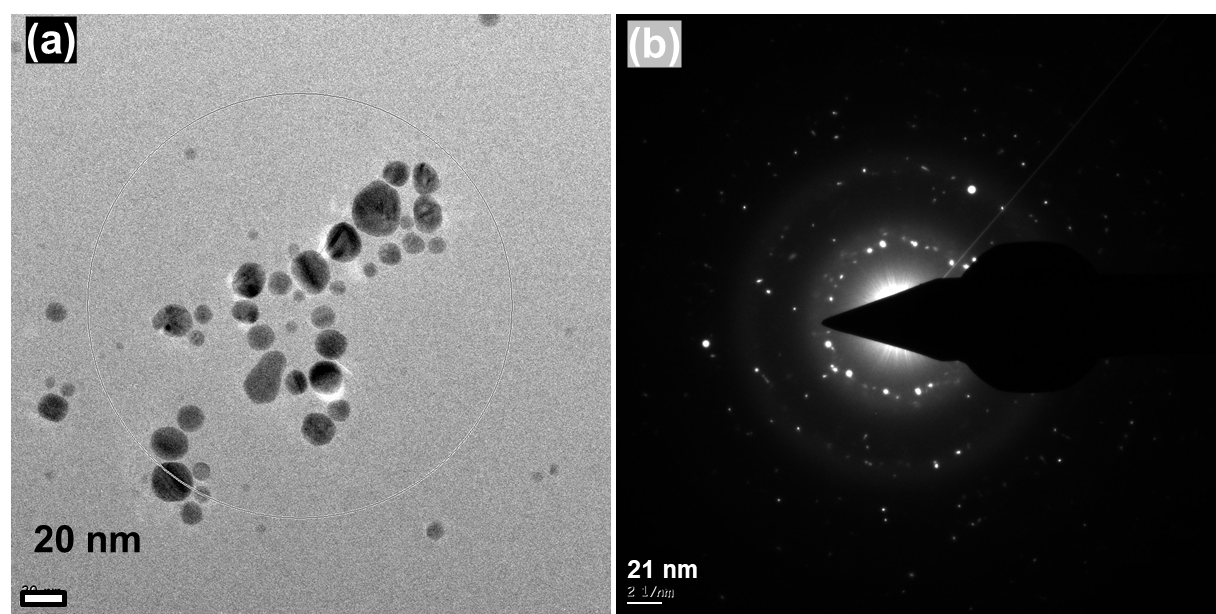


**Figure S3.** (a) HR-TEM image of AgNPs (b) A selected area electron diffraction pattern of AgNPs.


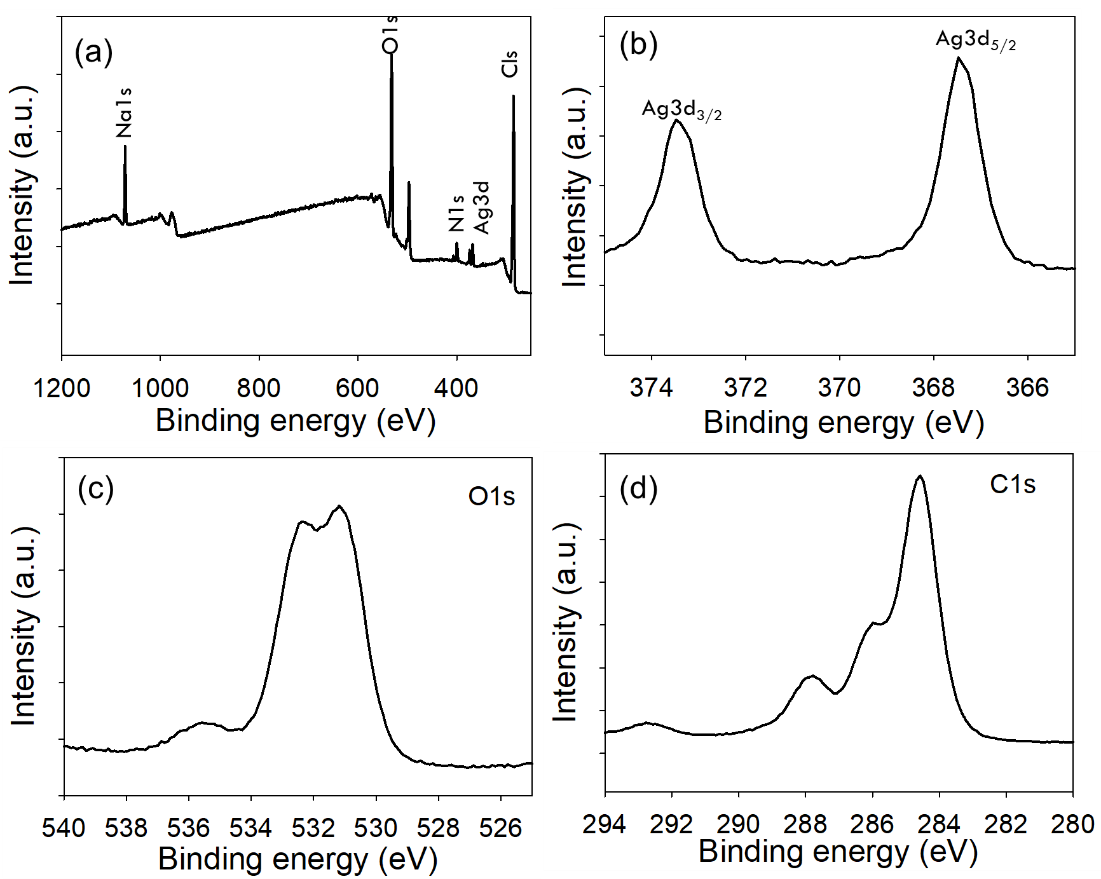


**Figure S4.** XPS spectral confirmation of metallic Ag and biomolecules at the surface for capping: (a) Survey spectrum that identifies elemental nature, (b) Ag 3d spectrum with peaks showing metallic Ag, (c) O 1s spectrum showing oxygen functional groups, and (d) C 1s spectrum showing the role of biomolecules in stabilization.


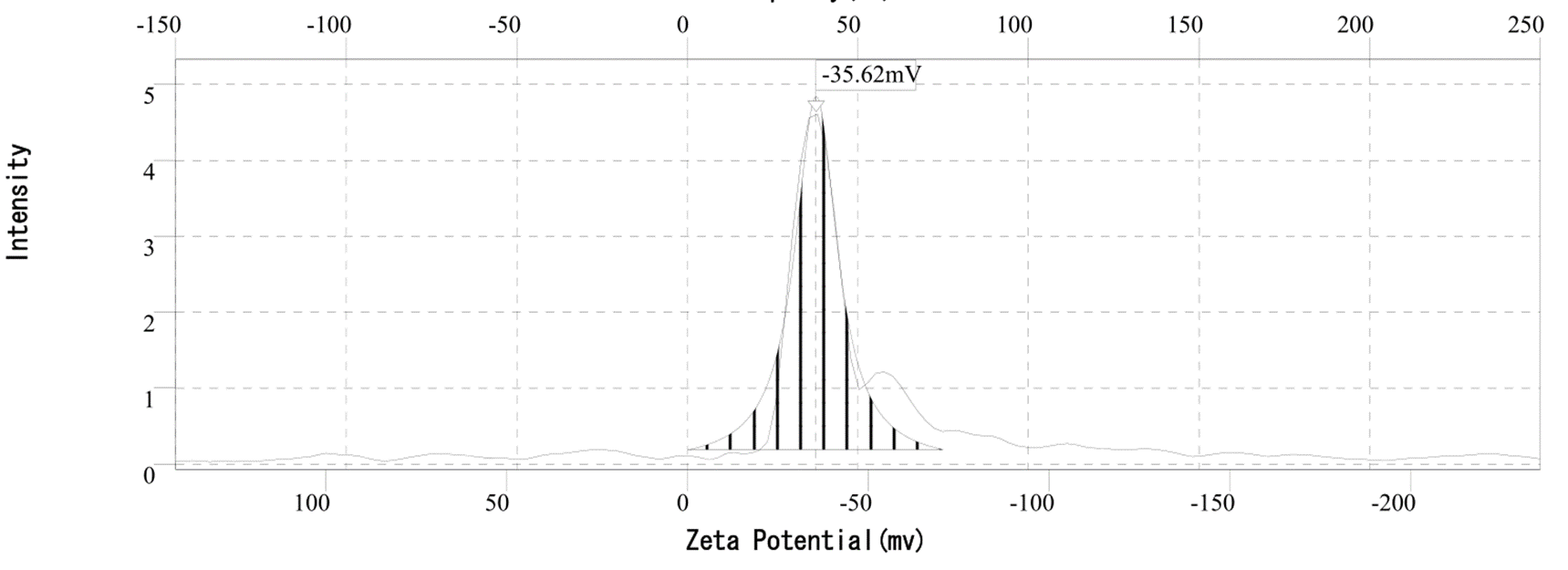


**Figure S5.** Zeta potential measurement of the AgNP colloidal solution.


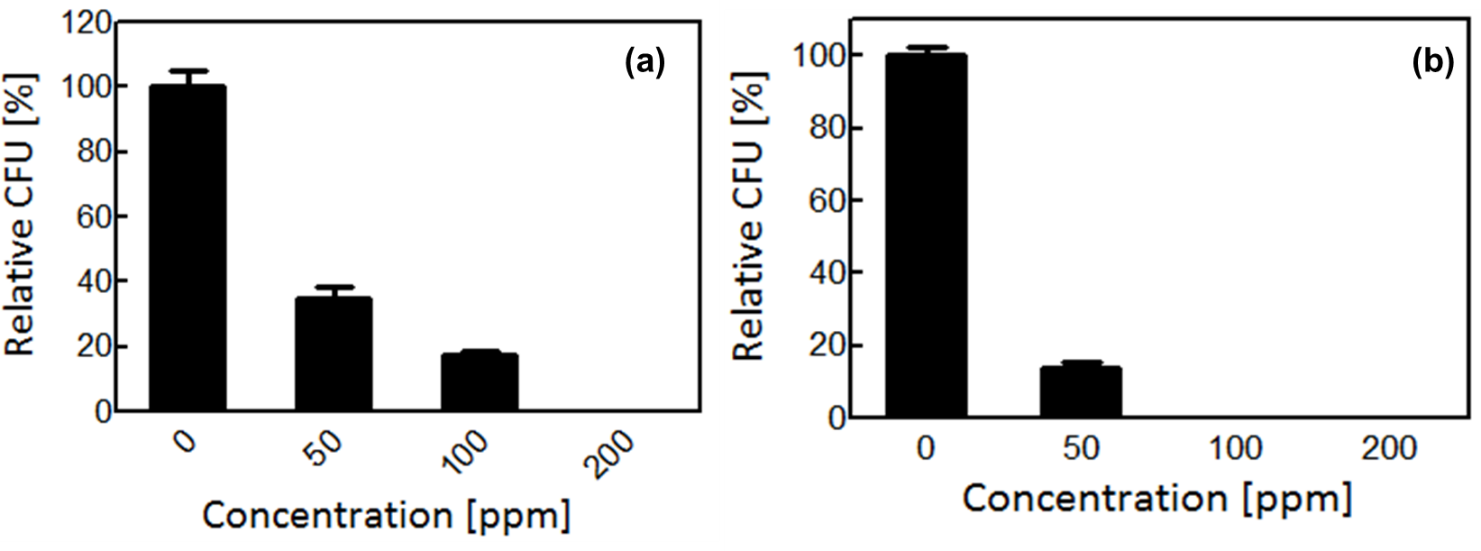


**Figure S6.** Bactericidal activity of AgNPs (a) Relative colony forming units of *E. coli* at different concentrations of AgNPs (b) Relative colony forming units of *S. aureus* at different concentrations of AgNPs.


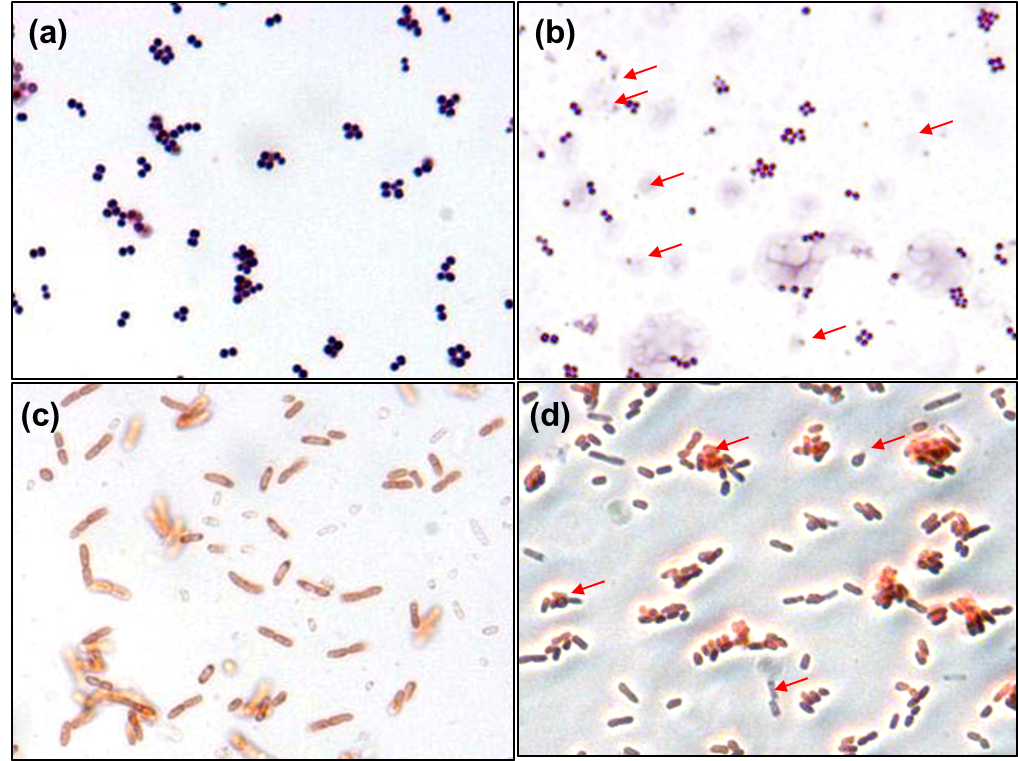


**Figure S7.** Gram staining images (a and b) before and after staining of *S. aureus* cells, (c and d) before and after staining of *E. coli* cells.


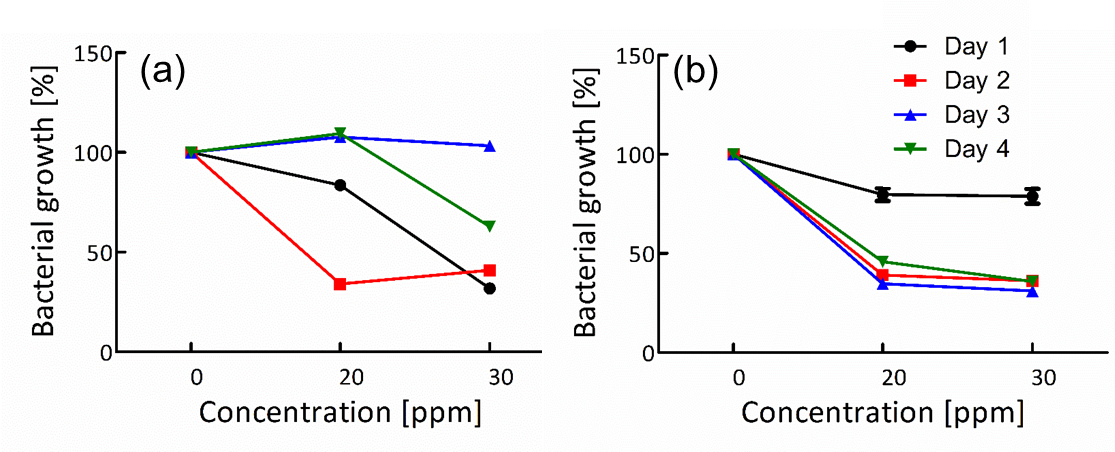


**Figure S8.** Serial passage assay evaluating the potential for bacterial adaptation to biosynthesized AgNPs, in which *E. coli* and *S. aureus* were cultured through consecutive growth cycles under sub-lethal AgNP exposure (a) *E. coli* exhibited a gradual recovery in viability over successive passages, suggesting an adaptive response. (b) In contrast, *S. aureus* showed no significant recovery during the assay period.

**
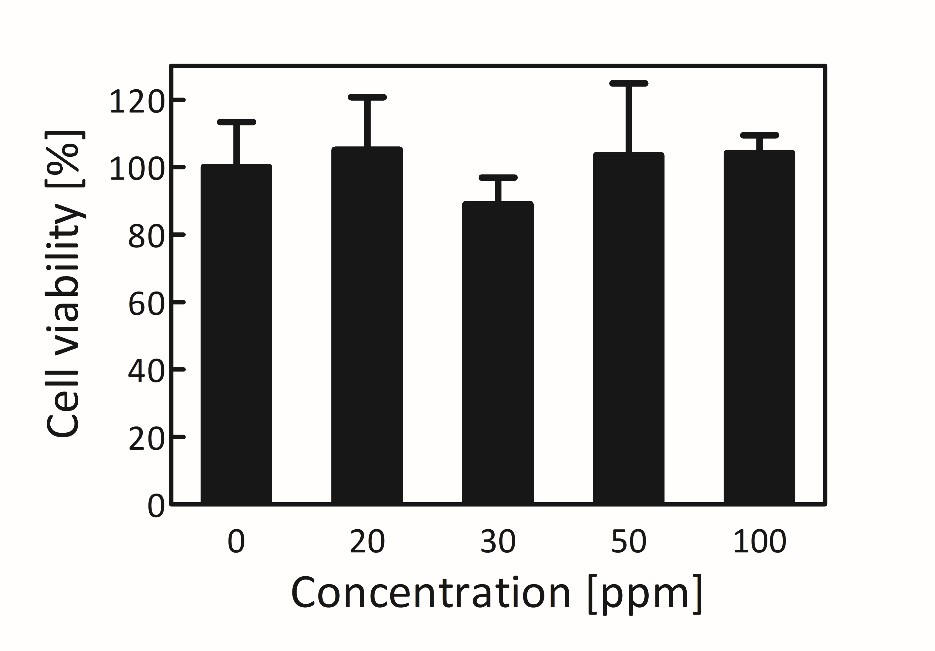
**

**Figure S9**. Cytotoxicity of AgNPs at varying concentrations against mouse embryonic fibroblast cells.

**Table S1. The phytochemical composition of radish roots contains a variety of compounds (flavonoids, glucosinolates, phenolic acids, carotenoids and others).**

| **Component** | **Examples** | **Reference** |
| --- | --- | --- |
| **Water** | ~96.1% | (Duy et al., 2019) |
| **Sugars and dietary fibers** | ~3.% | (Banihani, 2017) |
| **Proteins** | ~0.73% | (Duy et al., 2019) |
| **Flavonoids** | Quercetin, Kaempferol, Myricetin | (Miean and Mohamed, 2001) |
| **Phenolic Acids** | p-Coumaric acid, Ferulic acid, Caffeic acid | (Jing et al., 2014) |
| **Glucosinolates** | Glucoraphasatin, Glucoraphenin | (Gamba et al., 2021) |
| **Terpenes** | Linalool, Eugenol | (Jia et al., 2023) |
| **Other Phytochemicals** | Anthocyanins, β-carotene, L-ascorbic acid | (Keyata et al., 2021) |
| **Amino Acids** | Glutamic acid, Aspartic acid | (Cai et al., 2024) |
| **Organic Acids** | Oxalic acid, Malic acid | (Cai et al., 2024) |

**References**

Banihani, S.A. (2017). Radish (Raphanus sativus) and Diabetes. *Nutrients* 9(9). doi: 10.3390/nu9091014.

Cai, X., Zhu, K., Li, W., Peng, Y., Yi, Y., Qiao, M., et al. (2024). Characterization of flavor and taste profile of different radish (Raphanus Sativus L.) varieties by headspace-gas chromatography-ion mobility spectrometry (GC/IMS) and E-nose/tongue. *Food Chemistry: X* 22**,** 101419. doi: <https://doi.org/10.1016/j.fochx.2024.101419>.

Duy, H.H., Ngoc, P.T.K., Anh, L.T.H., Dao, D.T.A., Nguyen, D.C., and Than, V.T. (2019). In Vitro Antifungal Efficacy of White Radish (Raphanus sativus L.) Root Extract and Application as a Natural Preservative in Sponge Cake. *Processes* 7(9)**,** 549.

Gamba, M., Asllanaj, E., Raguindin, P.F., Glisic, M., Franco, O.H., Minder, B., et al. (2021). Nutritional and phytochemical characterization of radish (Raphanus sativus): A systematic review. *Trends in Food Science & Technology* 113**,** 205-218. doi: <https://doi.org/10.1016/j.tifs.2021.04.045>.

Jia, X., Yu, P., An, Q., Ren, J., Fan, G., Wei, Z., et al. (2023). Identification of glucosinolates and volatile odor compounds in microwaved radish (Raphanus sativus L.) seeds and the corresponding oils by UPLC-IMS-QTOF-MS and GC × GC-qMS analysis. *Food Research International* 169**,** 112873. doi: <https://doi.org/10.1016/j.foodres.2023.112873>.

Jing, P., Song, L.-H., Shen, S.-Q., Zhao, S.-J., Pang, J., and Qian, B.-J. (2014). Characterization of Phytochemicals and Antioxidant Activities of Red Radish Brines during Lactic Acid Fermentation. *Molecules* 19(7)**,** 9675-9688.

Keyata, E.O., Tola, Y.B., Bultosa, G., and Forsido, S.F. (2021). Phytochemical contents, antioxidant activity and functional properties of Raphanus sativus L, Eruca sativa L. and Hibiscus sabdariffa L. growing in Ethiopia. *Heliyon* 7(1)**,** e05939. doi: <https://doi.org/10.1016/j.heliyon.2021.e05939>.

Miean, K.H., and Mohamed, S. (2001). Flavonoid (myricetin, quercetin, kaempferol, luteolin, and apigenin) content of edible tropical plants. *J Agric Food Chem* 49(6)**,** 3106-3112. doi: 10.1021/jf000892m.
